# Supplementary material for: Genome-wide mapping of cyclic AMP receptor protein binding in Enteroaggregative Escherichia coli reveals targeting of virulence-associated genes
Source: bioRxiv. 2025 Aug 11:2025.05.09.652842. Preprint. [Version 2] doi: 10.1101/2025.05.09.652842 (PMC12363807; doi:10.1101/2025.05.09.652842)
Supplement: Supplement 1 [file media-1.pdf]

Table S1: Strains, plasmids and oligonucleotides used

Strains

Strains	Characterisations	Source
EAEC 042	Wild type, prototype strain, Sm^R, Tet^R, Cm^R, Diarrhoeagenic in volunteers, expresses AAF/II, biofilm positive, harbours pAA2 plasmid.	(1)
E. coli M182	Δ*(codB-lacI)3, galK16, galE15(GalS),* λ^-*, e14-, relA1, rpsL150(strR), spoT1.*	(2)
E. coli M182 **Δ***crp*	Δ*(codB-lacI)3, galK16, galE15(GalS),* λ^-*, e14-, relA1, rpsL150(strR), spoT1 and* Δ*crp*.	(3)

Plasmids

Plasmids	Description	Source
pRW50	Low-copy- number *lacZ* expression vector, allows promoter fragments to be cloned using *Eco*RI and *Hin*dIII sites as fusions to *lacZ* transcription. Carries tetracycline resistance gene.	(4)
pRW224	A pRW50 derivative which allows promoter fragments to be cloned using *Eco*RI and *Hin*dIII sites as fusions to *lacZ* transcription, carries tetracycline resistance gene.	(5)
pSR	Supercoiled small plasmid with 2.6 kb with *Eco*RI and *Hin*dIII sites for inserting the desired promoter fragment which will be located upstream of a λ*oop* terminator site. Carries ampicillin resistance gene.	(6)
pD	pBR322 derivative. Carries ampicillin resistance gene.	(7)
pDCRP	pD derivative carrying the *crp* gene. Carries ampicillin resistance gene.	(7)
pDCRP $AR1^-$	A pDCRP derivative carrying a substitution in CRP activating region AR1 (HL159).	(7)
pDCRP $AR2^-$	A pDCRP derivative carrying a substitution in CRP activating region AR2 (KE101).	(7)
pDCRP $AR1^-$ & 2^-	A pDCRP derivative carrying substitutions in CRP activating regions AR1 (HL159) and AR2 (KE101).	(7)

Oligonucleotide primers

Oligo name	Sequence (5`-3`)**	Use
Primers used to sequence pRW50/pRW224 constructs		
pRW50 F	CCCTGCGGTGCCCCTCAAG	Primer for sequencing in the forward direction, binding upstream of the *Eco*RI site of pRW50/ pRW224.
pRW224 R	GGCGATTAAGTTGGGTAACGCCAGGG	Primer for sequencing in the reverse direction, binding downstream of *Hin*dIII site in pRW224.
pRW50 R	GCAGGTCGTTGAACTGAGCCTGAAATTCAG	Primer for sequencing in the reverse direction, binding downstream of *Hin*dIII site in pRW50.
Primers used to generate fragments used for EMSA experiments		
EC042_0225 F	GGGGGGAATTCAACTCGAATAAAGAAAAGGGTGTG	Forward primer to amplify EC042_0225 fragment.
EC042_0225 R	GGGGGAAGCTTATGGGGTTGGCATTTATG	Reverse primer to amplify EC042_0225 fragment.
EC042_0414 F	GGGGGGAATTCATGTTGCAATCTTCTGCTGACAAAGC	Forward primer to amplify EC042_0414 fragment.
EC042_0414 R	GGGGGAAGCTTTTAACTTATAATTAAGAGAAAAAAC	Reverse primer to amplify EC042_0414 fragment.
p0536_100 F	GGGGGAATTCTGGTGTTTACGCTTACACCAGACA	Forward primer to amplify p0536 fragment and it’s derivatives.
p0536_100 R	GGGGAAGCTTTACAGTTTTCATGCATTTTTTCTC	Reverse primer to amplify p0536 fragment and it’s derivatives.

EC042_3143 F	GGGGGGAATTCATGTCCGTTTGCGGACAAGCAATAG	Forward primer to amplify EC042_3143 fragment.
EC042_3143 R	GGGGGAAGCTTGGGAAACCGGTGTTTTGAAAACAGT	Reverse primer to amplify EC042_3143 fragment.
EC042_3975 F	GGGGGGAATTCAGATTCGGTTTTTCAGACCCCCATC	Forward primer to amplify EC042_3975 fragment.
EC042_3975 R	GGGGGAAGCTTATAAGAGTTATTCAAAATATTTTGT	Reverse primer to amplify EC042_3975 fragment.
p*kpsM*_201 F	GGGGGGAATTCTAATTACCTTCGGGATTATTGATG	Forward primer to amplify p*kpsM*_201 fragment.
p*kpsM*_201 R	GGGGGAAGCTTCTGTTTCAAACCAGAACGCACGCG	Reverse primer to amplify p*kpsM*_201 fragment.
p*mchA*_600 F:	GGAATTCCGCTGGTATTGCGGAAATCAG	Forward primer used to amplify a 619 bp "p*mchA*_600" used for generating the p*mchA* promoter construct.
p*mchA* R	GGGAAGCTTTACGTTTTCGCATTAAAAAAGTCCG	Reverse primer used to amplify a 619 bp "p*mchA* intermediate" used for generating the p*mchA* promoter construct.
p*mchA*_200 SDM F:	GTAGGTTGGCATAACTGTC	Forward primer used to generate the pmchA promoter construct by site directed mutagenesis (SDM) using the "p*mchA* intermediate" as template.
p*mchA*_200 SDM R:	GAATTCAGATCTAGCGTG	Reverse primer used to generate the pmchA promoter construct by (SDM) using the "p*mchA* intermediate" template.
p*mchA*_200 F:	GGAATTCGTAGGTTGGCATAACTGTCCTG	Forward primer used to amplify p*mchA* promoter construct, used with p*mchA* R to generate 213 bp fragment.
virK F	GGGGGGAATTCTCATGTTTTCCGGCAATTGAGATAC	Forward primer to amplify *virK* fragment.

virK R	GGGGGAAGCTTTTTCGGTACTCAGAGCGTTTTTTAC	Reverse primer to amplify *virK* fragment.
Primers used to generate mchA *promoter derivatives*		
p*mchA* 37G F	TGCATAAGCT**g**ACATAAAAATAATATTAATTATG	Forward primer to introduce 37G mutation in the p*mchA* promoter construct by SDM.
p*mchA* 37G R	CAAAAATAACCAAGTATCTTATC	Reverse primer used to introduce 37G mutation into the p*mchA* promoter construct by SDM.
p*mchA* p11G F	TAATTATGTT**g**TCTTAGCGATAGAAAAATAAAC	Forward primer to introduce 11G mutation in the p*mchA* promoter construct by SDM
p*mchA* p11G R	ATATTATTTTTATGTGAGCTTATGC	Reverse primer used to introduce 11G mutation into the p*mchA* promoter construct by SDM.
p*mchA* p11A F	TAATTATGTT**a**TCTTAGCGATAGAAAAATAAAC	Forward primer to introduce 11A mutation in the p*mchA* promoter construct by SDM
p*mchA* p11A R	ATATTATTTTTATGTGAGCTTATG	Reverse primer used to introduce 11A mutation into the p*mchA* promoter construct by SDM
p*mchA* SOE 14A left arm F:	GGAATTCGTAGGTTGGCATAACTGTCCTGTAACTCATTGTTCAAAAAAGAAAGCTCCTACAAACTGTATCCTGTATCTC	Forward primer used to generate left arm of p*mchA* 14A promoter variant.
p*mchA* SOE 14A left arm R:	GCTTATGCACAAAAATAACCAAGTATCTTATCTTCTGGTAGAGATACAGGATACAGTTTGTAGGAG	Reverse primer used to generate left arm of p*mchA* 14A promoter variant.
p*mchA* SOE 14A right arm F:	GATACTTGGTTATTTTTGTGCATAAGCTCACATAAAAATAATATTAATTAT**a**TTTTCTTAGCGATAGAAAAATAAACAGGTAAGCAG	Forward primer used to generate right arm of p*mchA* 14A promoter variant.

p*mchA* SOE 14A right arm R	GGGAAGCTTTACGTTTTCGCATTAAAAAAGTCCGTCTTCTGCTTACCTGTTTATTTTTCTATCGCTAAG	Reverse primer used to generate right arm of p*mchA* 14A promoter variant.
Primers used to generate 0536 *promoter derivatives*		
p0536_103 17G F	GCAACGCAGAT**g**AATTCTTATAAC	Forward primer to introduce 17G mutation in the p0536 promoter construct by SDM.
p0536_103 17G R	AATTTAATCGAAATCTACGC	Reverse primer to introduce the 17G mutation in the p0536 promoter construct by SDM
p0536_104 25G F	ATTAATTCTT**g**TAACAACGTTTTACG	Forward primer to introduce 25G mutation in the p0536 promoter construct by SDM.
p0536_104 25G R	CTGCGTTGCAATTTAATC	Reverse primer to introduce 25G mutation in the p0536 promoter construct by SDM.
p0536_105 11G F	ATGGCGGCGT**g**GATTTCGATT	Forward primer to introduce 11G mutation in the p0536 promoter construct by SDM.
p0536_105 R 11G	TTGTGTGATGTAAAGCGC	Reverse primer to introduce 11G mutation in the p0536 promoter construct by SDM
p0536_106 1G F	AGATTTCGAT**g**AAATTGCAACGCAGATTAATTC	Forward primer to introduce 1G mutation in the p0536 promoter construct by SDM.
p0536_106 1G R	ACGCCGCCATTTGTGTGA	Reverse primer to introduce 1G mutation in the p0536 promoter construct by SDM
p0536_107 38T 36A F	GATCGAATTCTGGTGTTTACGCTTACACCAGACAAAAATG**t**G**a**TTTACATCACACAAATGGCGGCGTAGATTTCGATTAAATTGCAACGCAGATTAATTCTTATAACAACG	Forward primer to introduce 38T 36A mutations in the p0536 promoter construct by PCR.

p0536_107 38T 36A R	GATCAAGCTTTACAGTTTTCATGCATTTTTTCTCTTTCAAAGTAAGTCACATATTTGTTTCTATAAGCAACGTAAAACGTTGTTATAAGAATTAATCTGCG	Reverse primer to introduce 38T 36A mutations in the p0536 promoter construct by PCR.
Primers used to generate kpsM *promoter derivatives*		
p*kpsM*_440 F	GGGGGGAATTCTTATTAATAGTTGCAATAAATCA	Forward primer to amplify p*kpsM*_440 and P2- fragments.
p*kpsM*_440 R	GGGGGAAGCTTTGTTCACCGAGAAACTATTT	Reverse primer to amplify p*kpsM*_440 and P1- fragments.
p*kpsM* P1- F	GGGGGGAATTCTTATTAATAGTTGCAATAAATCATTGAGTAACAATTGATAGGCCAAAACATATAGGATAATTCTTGTGTGATCTGT**g**TTTTGTGTAGC	Forward primer to introduce 13G mutation in p*kpsM*_440 P1- and P1-P2- fragments.
p*kpsM* P2- R	GGGGGAAGCTTTGTTCACCGAGAAACTATTTCCCTATTTAAAATTCACTCGTGTACTTCTTATTTATATCTACAGCCCCCTCTTTACAGTCATATTTGTGATTTA**c**ATCACATTTA	Reverse primer to introduce 245G mutation p*kpsM*_440 P2- and P1-P2- fragments.

** Underlined bases represent the site for restriction enzymes *Eco*RI or *Hind*III. Base changes introduced by primers are in lower case and in bold.

Table S2: List of all CRP sites in *Escherichia coli* 042 as determined by ChIP-seq

Peak centre [a]	Score [b]	Annotated gene [c]	*E. coli* homologues [d]	Position relative to TSS [e]	Motif identified [f]	P-value [g]	Regulated by CRP? [h]	Gene product [i]
Targets on the 042 chromosome								
4488945	19348	EC042_4210	*udp*	-81.5	GGTGATGGGTATCACG	4.54E-05	Y	Uridine phosphorylase
4149109	17137	EC042_3905	*mtlA*	-224.5	TGTGATTCAGATCACA	4.16E-08	Y	Mannitol-specific PTS system EIICBA component
48719	16242	*fixA*	*fixA*	-204.5	GGTGATCTATAAAACA	1.59E-03	Y	Probable electron transfer flavoprotein subunit of oxidoreductase
2531800	12889	EC042_2405	*mtlD*	-101.5	ATTGATCGCCCTCACA	1.14E-04	Y	Mannitol dehydrogenase family protein
3838726	12183	*tsgA*	*ppiA*	-129.5	CGTGATGAGAATCACT	4.95E-05	Y	Peptidyl-prolyl cis-trans isomerase
2993024	8657	*raiA*	*raiA*	-70.5	TGAGATTTCCATCACA	1.74E-05	Y	Ribosome-associated inhibitor A
2784492	7180	*ptsH*	*ptsH*	-326.5	TGTGGCCTGCTTCAAA	4.95E-05	Y	Phosphocarrier protein Hpr
1250181	7061	EC042_1182	*ptsG*	-76.5	TGTGATCCAGATCACA	1.40E-09	Y	PTS glucose transporter subunit IIBC
3356318	6783	EC042_3143	Uncommon	38.5	TGTGATCTACAACACG	1.50E-05	-	Hypothetical protein
3350600	5944	*epd*	*epd*	-202.5	TCTGACTCACATCACA	9.63E-06	Y	D-erythrose 4-phosphate dehydrogenase
2529223	4498	*fruB*	*fruB*	-167.5	TTTGATGTGCTGCACA	3.81E-05	N	Multiphosphoryl transfer protein
4560899	4155	*fdhD*	*fdhD*	39.5	TGTGACAAATGTCACA	1.64E-06	N	Putative formate dehydrogenase accessory protein
5218617	4089	EC042_4877	*deoC*	-119.5	TTTGAACCAGATCGCA	1.23E-05	Y	Deoxyribose-phosphate aldolase
2151336	4034	*ftnB*	*flhD*	-40.5	TTTGATTTATCTTGCA	1.69E-03	Y	Transcriptional activator FlhD
2497609	3901	*cdd*	*cdd*	-67.5	TGAGATTCAGATCACA	9.11E-06	Y	Cytidine deaminase
4954732	3900	*fxsA*	*aspA/fxsA*	-145.5	CGTAATCTGGATCACT	2.93E-04	Y	Aspartate ammonia-lyase/ membrane protein FxsA
4590079	3873	*glpF*	*glpF*	244.5	TGCGCTTGTCGAAACA	4.23E-03	Y	Glycerol uptake facilitator protein

3377662	3657	*ansB*	*ansB*	-114.5	TTAGAGGCAGGTAACA	1.85E-03	Y	L-asparaginase 2
2535535	3525	*spr*	*mepS*	-119.5	TGTGCGTTAGTCCACA	4.95E-05	N	Lipoprotein
3190075	3516	*sdaC*	*sdaC*	-168.5	TGGGATCAAGATCACT	7.25E-05	N	Serine transporter
3719403	3355	*elbB*	*elbB*	-58.5	AATGCTACGCATCACA	1.50E-03	N	Enhancing lycopene biosynthesis protein 2 (sigma cross-reacting protein 27A)
2073958	3353	EC042_1989	*manX*	125.5	CTCGATCAGACGCACA	5.52E-04	Y	Mannose-specific PTS system EIIAB component
5094768	3314	EC042_4748	In K-12	-67.5	TGCGACAATCATCACA	5.12E-06	-	PTS system EIIA component
4398875	2967	*rbsD*	*rbsD*	-91.5	TTCGAGGTTGATCACA	2.10E-05	Y	Putative ribose transport (metabolism protein)
2034392	2844	EC042_1961	*gapA*	-87.5	TGTGACGAGCATCACG	1.36E-06	Y	Glyceraldehyde 3-phosphate dehydrogenase A
3382409	2794	*nupG*	*nupG*	40.5	TGTGAGGAAATTAACA	1.18E-04	Y	Nucleoside permease NupG
2430107	2264	EC042_2320A	*yegQ*	834.5	TTAGAAACCGATCACA	4.49E-04	N	tRNA 5-hydroxyuridine modification protein YegQ
4682235	2044	*purH*	*purH*	-352.5	TGTGAATCACTTCACA	3.28E-06	N	Bifunctional purine biosynthesis protein
2795558	1964	*ucpA*	*ucpA*	-82.5	TGCGGATCAGCTCACT	4.98E-04	N	SDR family oxidoreductase UcpA
2773567	1953	EC042_2614	*yfeC*	-88.5	AGTTATTCATGTCACG	9.67E-04	N	Putative DNA-binding transcriptional regulator
494392	1853	*tsx*	*tsx*	-151.5	ATCGATTGCGTTCACG	1.55E-03	Y	Nucleoside-specific channel-forming protein Tsx
2975867	1701	EC042_2790	*patZ*	-63.5	TCTGGGTAGCATCACA	2.27E-04	Y	Protein lysine acetyltransferase
1190486	1700	*rne*	*ycdZ*	-241.5	TGCAACCCGCAGCCCG	5.37E-03	Y	DUF1097 domain-containing protein
719805	1585	*rnk*	*rnk*	-129.5	AGTGATTTGCGTCACA	7.43E-06	N	Nucleoside diphosphate kinase regulator
4801045	1563	*proP*	*proP*	-216.5	TGTGAAGTTGATCACA	2.69E-07	Y	Proline (betaine transporter)
3615779	1543	*uxaC*	*uxaC*	-99.5	CGTGAGATAGATCAAT	3.04E-04	Y	Glucuronate isomerase
2877210	1487	*xseA*	*xseA*	-7.5	TTTGATCTCGCTCACA	2.63E-06	Y	Exodeoxyribonuclease VII large subunit
1336252	1427	EC042_1262	*dhaR*	-94.5	TGTGATCACGCCCGCA	1.30E-05	N	PTS-dependent dihydroxyacetone kinase operon regulator (sigma-54 dependent transcriptional regulator)

1866407	1300	*malX*	*uidR*	-83.5	TGTGATTTATGCCTCA	1.81E-04	N	*uid* operon repressor (TetR-family transcriptional regulator)
459356	1136	EC042_0411	*yaiZ*	71.5	TGTTGTCTACGCCACA	1.88E-04	N	Putative membrane protein
3951363	1108	*rpoH*	*rpoH*	-107.5	CGTGATTTTATCCACA	9.67E-05	Y	RNA polymerase sigma-32 factor RpoH
3152883	1050	EC042_2961	In K-12	-33.5	TGTGTCGGCGGTCAAT	1.21E-03	-	Putative FAD-dependent oxidoreductase
732988	1014	*cspE*	*cspE*	-103.5	CGCGACTTTTATCACT	2.53E-04	Y	Cold shock-like protein
3293513	1002	EC042_3089	*ygfK*	-271.5	AGTTCTCTTTATCATA	9.61E-03	N	Pyridine nucleotide-disulfide oxidoreductase
2480548	911	EC042_2359	*btsS*	-95.5	CGTGAGTACGATCACT	8.56E-05	N	Two-component system sensor kinase
3459052	894	*kpsM*	Uncommon	-492.5	TGTGATTTATATCACA	2.69E-07	-	Polysialic acid transport permease protein
736066	860	*lipA*	*lipA*	-119.5	CCTGAAAGCAGCCAAA	6.96E-03	N	Lipoyl synthase
3012994	857	EC042_2821	In K-12	-175.5	TCCGCTCTGGCTCATA	1.91E-03	-	Conserved hypothetical protein
3645715	857	*garD*	*garD*	-177.5	TGCGCGCTAAAGCACA	3.33E-05	N	D-galactarate dehydratase pseudo
5030357	827	EC042_4698	*ytfJ*	-109.5	TGCGGTCAAGCGCACA	3.81E-05	N	YtfJ family protein
5116358	827	*msbB2*	Uncommon	-233.5	TGTACCAGTCACCACA	1.37E-03	-	Virulence protein
1381630	819	EC042_1347	*adhE*	-638.5	TGTGATGAAAGCCTGT	8.09E-03	N	Aldehyde-alcohol dehydrogenase
3878947	818	*pckA*	*pckA*	-69.5	TATGAGCCTTGTCGCG	9.07E-04	Y	Phosphoenolpyruvate carboxykinase [ATP]
2627362	785	*glpT*	*glpT*	-118.5	TGTGAATTACCGCACA	1.01E-05	Y	Glycerol-3-phosphate transporter
5192683	765	EC042_4852	*btsT*	-88.5	TGTTGCCAGAGTTACG	6.96E-03	Y	Putative carbon starvation protein
3638072	758	*tdcA*	*tdcA*	-71.5	TGTGCGACCACTCACA	3.48E-05	Y	Tdc operon transcriptional activator
2769499	755	*nupC*	*nupC*	-103.5	TTTGAAGCTGGTCACA	1.36E-05	Y	Nucleoside permease NupC
3747290	727	EC042_3523	*yhcN*	-49.5	TGTGATATGGGTCACG	8.60E-07	N	Conserved hypothetical protein
2622643	712	EC042_2478	*lysR/pseudo gene*	-71.5	TGTGATGTACTTTGCA	1.05E-04	N	LysR-family transcriptional regulator (partial)/ MFS transporter Pseudo
3335320	692	*serA*	*serA*	-199.5	CGTGACACATGTCACC	8.56E-05	Y	Phosphoglycerate dehydrogenase
3231340	646	EC042_3028	In K-12	-126.5	CGTGACCCAGGTCACA	1.67E-07	-	Hypothetical protein
1177277	639	EC042_1101	*putA/putP*	6.5	TGTGTGCTCGATCTCA	2.44E-04	Y	Bifunctional protein PutA/sodium proline

								symporter (proline permease) PutP
1827122	631	*uidA*	*mlc*	-170.5	CGTGATATAGATCGCA	2.34E-06	Y	Protein Mlc (making large colonies protein)
4741151	625	EC042_4418	*pspG*	255.5	TGTGCGGATGATCACA	3.89E-06	N	Putative membrane protein
3421201	615	*mchA*	Uncommon	-79.5	TGTGAGCTTATGCACA	1.30E-05	-	Microcin activation protein
3931572	607	*gntK*	*gntK*	-121.5	TGTGAGCTACTTCAAA	9.11E-06	Y	Thermoresistant gluconokinase
228313	583	*gmhB*	*gmhB*	673.5	TGTGAATCACTTCACA	3.28E-06	N	D-**g**lycero-β-D-manno-heptose-1,7-bisphosphate 7-phosphatase
3832206	568	EC042_3619	In K-12	96.5	TGCAAAGGACGTCACA	1.88E-04	-	Hypothetical protein
4724645	552	*malE*	*malE*	-121.5	TGTGATCTCTGTTACA	3.18E-05	Y	Maltose transport system substrate-binding protein MalE
2416645	544	*mdtA*	*mdtA*	-432.5	TGCTGGCAGGATCGCA	6.31E-04	N	Multidrug resistance protein
1261443	508	*minD*	*bhsA*	-358.5	TGTGACCGGCGTTGTA	2.08E-03	Y	Putative exported protein
1314710	502	*dhaR*	*dadA*	-22.5	TGCGAGCCGGAACACC	3.77E-04	Y	D-amino acid dehydrogenase small subunit
2506198	500	*mglB*	*mglB*	-259.5	TGTGAAATCACTCACA	7.43E-06	Y	Galactose/glucose ABC transporter substrate-binding protein MglB
2205763	485	EC042_2120	*yedR*	-46.5	TGTGATAAAGGTCACA	1.95E-07	N	Putative membrane protein
3771532	442	*dusB*	*dusB*	-286.5	TGCGAGCGATGTCACA	2.78E-06	Y	tRNA-dihydrouridine synthase B
5027585	433	*cpdB*	*cpdB*	-74.5	AGTGAAGAATGCCACA	2.53E-04	Y	2',3'-cyclic-nucleotide 2'-phosphodiesterase
3101613	429	*ascG*	*ascF*	-93.5	GGTGACCGGTTTCACA	2.91E-05	Y	Arbutin-, cellobiose-, and salicin-specific PTS system EIIBC component
4388273	416	*atpI*	*atpI*	-252.5	CGTGCTTCAGATCACA	4.09E-06	N	ATP synthase protein I
467174	392	*aroM*	*aroM*	-208.5	AGGGATCTGCGTCACA	8.56E-05	N	AroM family protein
5020611	392	*cycA*	*cycA*	-187.5	TGTGAGCTGTTTCGCG	2.78E-05	N	D-serine, D-alanine, glycine transporter
5212600	384	*osmY*	*osmY*	-36.5	TGTTGCCAGGCTCAAA	7.96E-04	Y	Osmotically inducible protein Y
3676811	377	*deaD*	*deaD*	-22.5	TGTGAACCGGCTCAAA	1.12E-05	N	ATP-dependent RNA helicase (DEAD-box protein)
1711834	361	EC042_1653	*aslA*	-44.5	CGTGATTACGATCACA	1.85E-06	N	Putative sulfatase

4753197	338	EC042_4429	*yjcB*	-39.5	TGTGAACTATATCACA	3.14E-07	N	Putative membrane protein
3517716	329	EC042_3298	*yghB*	-75.5	TGCGTCCGGGATCAAG	5.90E-04	N	Putative membrane protein
461029	312	EC042_0414	Uncommon	72.5	TGTGCGCAAGATCACA	9.24E-07	-	Conserved hypothetical protein
4051417	311	EC042_3818	*yhjE*	-144.5	TGTGAAGCATTTCATA	2.83E-04	N	MHS family MFS transporter
4062646	308	*dctA*	*dctA*	-132.5	TTTGAGCTGGCTCGCA	2.10E-05	Y	C4-dicarboxylate transport protein
172	301	*thrA*	*thrA*	-218.5	ATTGACTTAGGTCACT	5.71E-04	N	Bifunctional aspartokinase I/homoserine dehydrogenase I
3899333	300	*malT*	*malT*	-131.5	TGTGACAGAGTGCAAA	7.56E-05	Y	Regulatory protein
4046225	299	*treF*	*treF*	-92.5	CGTGATCTACCGCACG	1.65E-05	N	Cytoplasmic trehalase
4272121	295	EC042_4013	*nepI*	-15.5	TGTGACGCATTTAACG	1.68E-04	N	Purine ribonucleoside efflux pump NepI
4412462	285	*hdfR*	*hdfR*	1050.5	CACGCAGGGGGTCGCG	7.32E-03	N	LysR-family transcriptional regulator (H-NS-dependent *flhD* regulator)
1475788	284	*insB*	*lapA*	140.5	GGTGACAGCAGGCTCA	2.08E-03	N	Putative membrane protein
3872334	283	*nudE*	*nudE*	-42.5	TGCGATATAGGACACG	1.18E-04	N	ADP compounds hydrolase
2079650	271	*pphA*	pseudo	-22.5	CGCGCTAAAGATCACA	2.78E-05	-	Conserved hypothetical protein (pseudogene)
4720171	269	*psiE*	*psiE*	-54.5	ATAGATCTCCGTCACA	7.70E-04	Y	Putative phosphate starvation-inducible membrane protein
4522062	267	*engB*	*engB*	950.5	TGTGATGGCTATTAGA	6.98E-04	N	Probable GTP-binding protein
4747362	265	*aphA*	*aphA*	-119.5	CCTGCTTTTCATCACA	1.81E-04	N	Class B acid phosphatase
3687170	262	*argG*	*argG*	-242.5	AGTGATCCACGCCACA	2.10E-05	Y	Argininosuccinate synthetase
3545591	258	*icc*	*icc*	-174.5	TGCTGGCTTGAACACA	2.63E-03	N	Repressor protein of division inhibition gene
2647958	252	*pmrD*	*pmrD*	-94.5	TGAGAAGTGAAACGGA	1.08E-02	N	Polymyxin B resistance protein
3510478	251	*hyb0*	*hyb0*	-77.5	TGCGCCATTTACCACA	1.81E-04	N	Hydrogenase-2 small chain
4217753	250	EC042_3975	Uncommon	-33.5	CGTGTATACGATAACA	2.48E-03	-	Hypothetical protein
2844005	245	*hyfA*	*hyfA*	-190.5	CGTGATCAAGATCACA	1.79E-07	Y	Hydrogenase-4 component A
3160614	242	EC042_2967	*ygcW*	-57.5	TGTGATCGTAATCACA	2.51E-07	N	Putative short chain dehydrogenase
147627	240	*gcd*	*gcd*	-69.5	TGTGATCGTCATCACA	7.11E-08	Y	Quinoprotein glucose dehydrogenase

4353137	229	*cat*	In K-12	-103.5	TGAGACGTTGATCGGC	2.71E-03	-	Chloramphenicol acetyltransferase
1344062	218	*adhE*	*ychH*	-230.5	CGTGATCCAAATCAAA	1.30E-05	Y	Putative membrane protein
2833992	209	EC042_2670	Uncommon	230.5	TGTGATACACAGCAAC	3.15E-04	-	Conserved hypothetical protein
4006081	208	EC042_3779	*dtpB*	-62.5	TGTAAACTTTTTCGCG	1.55E-03	N	Putative oligopeptide transporter
3784875	200	EC042_3554	*yhdZ*	928.5	CGCTACTGCCGCCAGA	8.30E-03	N	ABC transporter, ATP-binding protein
4752086	197	*ssb*	*ssb*	-89.5	CGGAACCGAGGTCACA	7.70E-04	N	Single-stranded binding protein
2941936	195	*glnB*	*glnB*	-40.5	TCTGCTAAACGTAACA	1.10E-03	N	Nitrogen regulatory protein p-II
167375	194	*sfsA*	*sfsA*	72.5	ATCGGGTGTGATCACA	2.34E-03	Y	Sugar fermentation stimulation protein
33766	193	*rihC*	*rihC*	-72.5	CGTGAAGTCGATTAAG	2.21E-03	N	Nonspecific ribonucleoside hydrolase (purine/pyrimidine ribonucleoside hydrolase)
3562340	187	EC042_3340	In K-12	-140.5	TTTGAGTTCGCACCCA	4.96E-03	-	Conserved hypothetical protein
2310574	176	*amn*	*amn*	186.5	TTGGGATGGTAGCACA	8.30E-03	N	AMP nucleosidase
4926886	174	*set1A*	Uncommon	48.5	ACTGACGGTTTTCCCA	6.61E-03	-	Enterotoxin 1
1444796	173	EC042_1404	*stfR*	-17.5	CTCTAACCACATAACG	1.65E-02	N	Phage side tail fiber protein
5007735	171	EC042_4670	*bsmA*	-53.5	TGTTACCTGGTACGCG	1.74E-03	N	Putative lipoprotein
608383	168	EC042_0536	Uncommon	-120.5	TGTGATGTAAAGCGCA	4.09E-06	-	Putative adhesin (not virulence)
4089215	168	*dppA*	pseudo	-500.5	AATGGTTTCTGTCACA	3.30E-03	-	Conserved hypothetical protein
3739372	164	*sspA*	*sspA*	-174.5	TGCGACCTTTGTGGTG	1.54E-02	N	Stringent starvation protein A
2678707	161	*lrhA*	*lrhA*	-178.5	CCTGAAGTAGATCACA	2.65E-05	N	NADH dehydrogenase operon transcriptional regulator
4448062	161	*aslB*	*aslB*	-277.5	AGTCCCCCCCCTCGCA	3.39E-03	N	Probable arylsulfatase-activating protein
3740611	156	*rplM*	*rplM*	-208.5	TGTGATTTGTGGCAGG	2.83E-04	Y	50S ribosomal subunit protein L13
3196847	155	*fucP*	*fucP*	548.5	TGCCACATCAATCGCA	7.21E-04	Y	L-fucose permease
5081393	155	EC042_4739	*ahr*	-129.5	TGCGAGCAAGCTGGCG	1.25E-03	N	NADPH-dependent aldehyde reductase Ahr
2272380	151	EC042_2206	*mtfA*	-181.5	TGTGATTTTTGTCACT	2.65E-05	N	DgsA anti-repressor MtfA
2068782	150	*manX*	*sdaA*	-155.5	ACGGATCTTCATCACA	1.06E-03	N	L-serine ammonia-lyase

1742072	146	*sotB*	*uxaB*	-83.5	CGCGATCCAGATCACA	6.52E-07	Y	Altronate oxidoreductase
4237814	145	EC042_3993	In K-12	-334.5	CGTGCTACCGGTCACG	3.98E-05	-	Phage integrase family (tyrosine-type recombinase/integrase)
1881643	144	EC042_1835	In K-12	-123.5	TTCGAGTCCACTCGGA	4.00E-03	-	Conserved hypothetical protein
968344	134	*clpS*	*ybiT*	-124.5	TGTGACAGATGTCGCT	8.90E-05	Y	ABC transporter ATP-binding protein
1038450	134	*focA*	*clpS*	135.5	TGCGATTGAGATGAAA	6.98E-04	N	ATP-dependent Clp protease adaptor protein
780664	122	*nagE*	*nagE/nagB*	-165.5	GGTGACAAAACTCACA	5.87E-05	Y	N-acetylglucosamine-specific PTS enzyme IIABC component/ Glucosamine-6-phosphate deaminase
4287533	122	*ivbL*	*ivbL*	-98.5	TGAGGGGTTGATCACG	3.51E-04	Y	*ilvBN* operon leader peptide
1072066	118	EC042_1043	*serC*	22.5	TGTGAACTCCGTCAGG	4.95E-05	Y	3-phosphoserine (phosphohydroxythreonine transaminase)
2576006	117	*napF*	*napF*	-124.5	CCCGATCGGGGTAAAA	3.89E-03	N	Ferredoxin-type protein
3417179	116	*mchS4*	Uncommon	13.5	TGTGATAATAATCACA	2.08E-06	-	Conserved hypothetical protein
2197691	115	EC042_2113	*yedP*	-110.5	TGTGACGCGCGTCACC	6.04E-06	N	Putative mannosyl-3-phosphoglycerate phosphatase
1825856	114	*mlc*	*mlc*	-97.5	TGTGATTAACAGCACA	2.21E-06	Y	Protein Mlc (making large colonies protein)
3364989	113	*galP*	*galP*	-71.5	TGTGATTTGCTTCACA	7.00E-07	Y	Galactose-proton symporter (galactose transporter)
461937	112	*phoA*	*phoA*	172.5	CGTGATTCTCTTAGCG	1.45E-03	N	Alkaline phosphatase
263739	111	EC042_0225	Uncommon	-80.5	TGTGAGCCGCATCACA	1.15E-07	-	Putative type VI secretion system protein
4116976	107	*malS*	*malS*	-61.5	TGAGAGTTGAATCTCA	1.03E-03	Y	Alpha-amylase
4160841	104	*lctP*	*lctP*	-46.5	GGAGATGAGCATCAGA	1.74E-03	N	L-lactate permease
5167710	103	EC042_4823	pseudo	14.5	TGATATATAGATAAGA	1.28E-02	-	Conserved hypothetical protein
544222	102	*maa*	*maa*	-86.5	TGTGATAAAGATCACA	1.34E-07	N	Maltose O-acetyltransferase
2164036	99	EC042_2071	*yecA*	-339.5	TTTGCAATCCGCTACA	8.30E-03	N	Conserved hypothetical protein
3353695	98	EC042_3140	*yggP*	472.5	TGTGATAATTGGCATG	1.91E-03	N	Zinc-binding dehydrogenase
263036	97	EC042_0224	Uncommon	62.5	TTTGGTGTATACCGAA	2.95E-03	-	Putative type VI secretion system protein

1868607	95	EC042_1802A	*malX/malI*	51.5	CGTGATCAAGATCACG	1.44E-06	Y	PTS maltose transporter subunit IIC/ *mal* regulon transcriptional regulator MalI
2214844	95	EC042_2130	Uncommon	814.5	AGTGCTGAATGTCACA	6.13E-05	-	Putative prophage protein
4101165	95	*cspA*	*cspA*	-32.5	ATCGCCGAAAGGCACA	3.21E-03	N	RNA chaperone/antiterminator CspA
2544035	94	EC042_2414	*yejG*	-147.5	TGCGGGCGTGGTCACG	5.87E-05	N	YejG family protein
1740383	90	*uxaB*	*uxaB*	-134.5	TGTGGCGCGGATCATG	3.77E-04	-	Putative membrane protein
2054834	90	*sdaA*	*yeaV*	-142.5	TGAGACAATCATCGCA	1.68E-04	N	BCCT family transporter YeaV
4812398	90	*melR*	*melR*	-63.5	TGCGAGTGGGAGCACG	5.63E-05	Y	Melibiose operon regulatory protein
379869	89	EC042_0340	In K-12	-186.5	TGTGAGCGATGCCGAA	2.53E-04	-	Conserved hypothetical protein
4530302	86	*typA*	*glnA*	-114.5	CGTGAAAGCGATCACA	6.68E-06	Y	Glutamine synthetase
4969712	85	EC042_4632	*yjeM*	-137.5	TCCGCTAAAGGCCACA	7.21E-04	N	Putative permease
1623270	82	EC042_1630	*ydcH*	-287.5	TGTGATGAATGTCACT	1.36E-05	N	YdcH family protein
2559209	81	EC042_2431	Uncommon	-285.5	TGATCCCCCATTAACG	2.94E-02	-	Putative prophage protein
2982786	81	*kgtP*	*kgtP*	-1116.5	TGCTCGCGCCGTCACG	7.21E-04	N	Alpha-ketoglutarate permease
2776365	79	EC042_2617	*fixA*-like family protein	-283.5	TGTGAAGTACCGAAGT	7.32E-03	N	Conserved hypothetical protein
3080457	78	EC042_2887	*yqaB*	-573.5	TCCGGGAGGATTCGAA	1.50E-02	N	Putative phosphatase
3332837	78	EC042_3122	*fau*	-118.5	GGTGAGCATGCTCGGT	4.83E-03	N	Conserved hypothetical protein
56171	77	*folA*	*folA*	-178.5	AGTGACGTAAATCACA	5.40E-06	N	Dihydrofolate reductase
937104	77	EC042_0886	*ybhQ*	-100.5	TATGCGCTGCGTCACA	1.33E-04	N	Putative membrane protein
1970534	77	EC042_1890	*infC*	-68.5	TGTGCGTTAGCTCGTG	1.85E-03	N	Translation initiation factor IF-3
4456964	77	*cyaA*	*cyaA*	-161.5	TGTTAAATTGATCACG	1.49E-04	Y	Adenylate cyclase
3346695	76	*mscS*	*mscS*	-102.5	TGCCAAATAGATCACA	1.88E-04	N	Small-conductance mechanosensitive channel
1418735	75	*stfR*	In K-12	-112.5	TGTTATGATACGCAGG	5.37E-03	-	Putative phage protein
4650149	75	*tufA*	*tufA*	-123.5	TCTGCCTATCAGCACC	2.79E-03	N	Elongation factor Tu
4167598	74	*secB*	*grxC*	-135.5	TGTGCTGTGCGTCAAT	1.95E-04	N	Glutaredoxin 3
4325818	71	EC042_4064	*tnaA*	-316.5	TGTGATTCGATTCACA	2.47E-06	Y	Tryptophanase

855435	69	*moaA*	*aroG*	2.5	TGTACATGGCTTCACA	9.67E-04	N	Phospho-2-dehydro-3-deoxyheptonate aldolase, Phe-sensitive
3407089	69	EC042_3190	Uncommon	62.5	CTCTCTGTCAGCCACT	3.81E-02	-	Conserved hypothetical protein
4734493	69	*plsB*	*plsB/dgkA*	8.5	CGTGGCCAGCCGGACA	2.71E-03	N	Glycerol-3-phosphate acyltransferase PlsB/ diacylglycerol kinase
150878	67	EC042_0128	*yadI*	-59.5	TTTGACGGCTATCACC	1.81E-04	N	PTS sugar transporter subunit IIA
4511085	67	*mobB*	*mob*	2473.5	AGTGCGAATGCTGACA	5.66E-03	N	Molybdopterin-guanine dinucleotide biosynthesis protein B
4664179	66	*htrC*	*yjaZ*	76.5	CTTGATGCTCATCAGG	8.23E-04	N	Heat shock protein C
3751263	64	*aaeR*	*aaeR*	-26.5	TGTGATCTAAATCACT	2.63E-06	Y	LysR-family transcriptional regulator
4646221	64	*murB*	*murB*	-244.5	CCTGGCGGCCGTAGCG	1.19E-02	N	UDP-N-acetylenolpyruvoylglucosami-ne reductase
1748762	63	EC042_1741	*ydeA*	-130.5	TGTTAACCCTGCAACA	3.30E-03	N	L-arabinose MFS transporter
816974	62	*sdhC*	*sdhC*	-145.5	AGTGTTTTGCATGACG	9.16E-03	Y	Succinate dehydrogenase cytochrome b-556 subunit
816974	62	*cydA*	*sdhC*	-134.5	TGTAACTTTTTTATCA	1.97E-02	Y	Succinate dehydrogenase cytochrome b-556 subunit
175121	61	*fhuC*	*fhuC*	-586.5	TGTGGTGACTGGCGCA	1.38E-04	N	Ferrichrome transport ATP-binding protein
3209376	59	*mltA*	*mltA*	-153.5	CGTGATCGGGGTAAAA	2.10E-04	N	Murein transglycosylase A
5129030	57	EC042_4783	*fecI*	-92.5	TCTGCTATTATTGACA	5.81E-03	N	RNA polymerase sigma factor
773400	56	EC042_0701	In K-12	-480.5	CTTGAACTCGCACACC	4.58E-03	-	Putative exported protein (pseudo)
2499767	56	EC042_2378	*preT*	-100.5	TGTGAATCCTTTCACA	1.36E-05	Y	Putative oxidoreductase
2095051	55	*torY*	*pphA*	470.5	TTTGATAAACCTTGCA	1.74E-03	N	Serine/threonine protein phosphatase 1
3892798	55	*gntT*	*gntT*	-190.5	TCTGGTGATTCTCAAA	2.08E-03	Y	High-affinity gluconate transporter
3966675	53	EC042_3740	In K-12	134.5	TGTGGGATACTTCCCG	1.10E-03	-	Putative acyltransferase
5101081	53	EC042_4755	In K-12	-80.5	TGTGACTGAGATCGCG	4.35E-06	-	Putative sugar kinase
3128509	52	*rpoS*	*nlpD*	-511.5	TGTGACCGTGGTCGCA	5.71E-07	Y	Murein hydrolase activator NlpD
2677252	50	*nuoA*	*nuoA*	-291.5	TGTGAAGCAATGGAAA	4.34E-03	N	NADH-quinone oxidoreductase subunit A

3932650	49	*gntR*	*gntR*	-42.5	GTTTAACACGGACGCA	2.54E-02	Y	Gluconate utilization operon repressor
242043	48	EC042_0209	In K-12	-417.5	CGTTCCCAACGGAACA	4.58E-03	-	Putative lipoprotein
3011421	48	EC042_2820	In K-12	-414.5	CGTAAAAAGCCGCAAA	1.06E-02	-	Integrase
5207449	48	EC042_4865	*yjjZ*	405.5	TGCGAGGGGGGGGACT	3.39E-03	N	Putative membrane protein
40727	46	*caiF*	*caiF*	-123.5	CAGGATTTAGCTCACA	1.80E-03	Y	Transcriptional activator
4359930	46	*aadA1*	Uncommon	102.5	TTTGTACGGCTCCGCA	4.71E-03	-	Aminoglycoside adenylyltransferase
3608027	45	EC042_3380	*ygjR*	48.5	TACGAACTGGATCACC	7.21E-04	N	Putative oxidoreductase
4374307	45	*tetR*	Uncommon	78.5	TTTGCGTGTCGTCAGA	1.33E-03	-	Tetracycline repressor
5230955	45	EC042_4888	ettA/sltY	-90.5	GTCGATCACCTTCGCA	1.06E-03	N	Energy-dependent translational throttle protein EttA/ murein transglycosylase
2917822	42	*EC042_2736*	*trmJ*	-33.5	CGCGCATCTTATCATA	5.10E-03	N	Putative RNA methyltransferase
3204810	42	*gcvA*	*gcvA*	-234.5	TGCGATTCAGACCATG	1.03E-03	N	Glycine cleavage system transcriptional activator
4674584	42	*hupA*	*hupA*	-50.5	TGGCATTTCCGTCGCA	2.02E-03	Y	DNA-binding protein HU-alpha
5121039	42	EC042_4775	In K-12	523.5	TGTCCGCTCTGGCACA	9.67E-04	-	Hypothetical protein
1976942	41	*msrB*	*yniA*	-126.5	CGTGATGAAAATCACA	1.74E-06	N	Fructosamine kinase family protein
4978451	41	*orn*	*orn*	1081.5	AGTGCTGTAAAGCACA	9.67E-05	N	Oligoribonuclease
5015959	41	EC042_4681	*rpsF*	-102.5	TGTCAGTACTATAACG	7.32E-03	Y	30S ribosomal subunit protein S6
125271	40	*nadC*	*ampD*	31.5	GTTGGCGCGCGCCGCG	7.89E-03	Y	N-acetyl-anhydromuramyl-L-alanine-amidase
3284051	40	EC042_3081	*ygeW*	-157.5	TGTGATCAACCCCACA	3.11E-06	N	Putative aspartate/ornithine carbamoyltransferase
841420	39	EC042_0769	*cydA*	-327.5	AGCGTGAAGGATAACG	1.80E-02	N	Cytochrome d ubiquinol oxidase subunit 1
851102	39	EC042_0773	Uncommon	-123.5	CTTGAAATAATTAACA	2.71E-03	-	Hypothetical protein
2051133	39	EC042_1966	*yeaQ*	-227.5	TTTGAACCGCGTCACT	1.28E-04	N	Putative transglycosylase associated protein
2507357	39	*galS*	*galS*	-84.5	CGTGAATCGAGTCACA	1.50E-05	Y	HTH-type transcriptional regulator GalS
4817898	39	*dcuB*	*dcuB*	-42.5	TGCTGAATAGATCACA	3.90E-04	N	Anaerobic C4-dicarboxylate transporter
3308819	37	EC042_3098	*ygfT*	-104.5	TGCGACTGAGTTCAAA	4.95E-05	N	Formate-dependent uric acid utilization protein YgfT

1915871	36	*lpp*	*ydhR*	-45.5	TGTAATACTTGTAACG	4.23E-03	N	Conserved hypothetical protein
4466034	35	EC042_4187	Uncommon	-7.5	CGAGGTGTTGATCACG	5.15E-04	-	Hypothetical protein
409039	34	EC042_0364	In K-12	-30.5	TGCGAGAGAGATCACA	2.21E-06	-	LysE-family translocator
2255919	34	EC042_2180	pseudo	556.5	TGTGAAGTATTTAAAA	7.21E-04	-	Putative prophage protein
4202532	33	EC042_3956	Uncommon	435.5	GGCGACATATGACGCG	5.10E-03	-	Putative prophage protein
4945596	33	EC042_4606	Uncommon	753.5	CTTGATGGAGCACAGA	3.39E-03	-	Hypothetical protein
2766013	32	EC042_2608	*glk/ yfeO*	-98.5	ATCGATCTGGGTCACA	7.25E-05	N	Glucokinase/Ion channel protein
3985355	32	EC042_3762	*gntR*	-129.5	ATCGACTCACGTCACA	2.93E-04	Y	GntR-family transcriptional regulator
4451798	32	*aslA*	*aslA*	-415.5	TGTGGCATAATAAACG	9.16E-03	N	Arylsulfatase
4005281	31	*uspA*	*uspA*	-148.5	GATCATCCGGGTCGCT	3.06E-02	N	Universal stress protein A
4290090	31	EC042_4031	*yidG*	-79.5	CGCGAGGGAGATCAAA	6.13E-05	N	Putative membrane protein
1865552	30	*uidR*	*uidA*	17.5	TGTGCTTCAGTCTGCA	2.15E-03	Y	Glucuronidase ß
2496355	30	EC042_2373	*yohj*	-57.5	TGTGATCGGTAGCACG	8.25E-06	N	Putative membrane protein
2782302	30	*zipA*	*zipA*	-70.5	TTTGCCGATTACCTCA	4.96E-03	N	Cell division protein
405459	29	EC042_0361	*yahK*	-161.5	TGTGATCTGCCACGAA	3.51E-04	N	NADPH-dependent aldehyde reductase YahK
526093	29	*ppiD*	*ppiD*	-77.5	CCCGTTTCTTGTCACA	4.11E-03	N	Peptidyl-prolyl cis-trans isomerase D
1139173	29	*cspG*	*yccA*	-26.5	TTTGCCGAAAGGCCCA	2.41E-03	N	Putative membrane protein
3485206	29	*glcC*	*glcC*	-10.5	TGTGCACGAGGTCCGG	1.80E-03	Y	Glc operon transcriptional activator
540752	28	EC042_0492	*ybaA*	-170.5	TGTTGGTTCTCTCGCA	1.64E-03	N	Conserved hypothetical protein
3186304	27	EC042_2990	*yqcC*	-182.5	TTGCTTCCTGCTCACA	2.44E-02	N	Conserved hypothetical protein
943255	26	EC042_0905	*cecR*	-179.5	TGCGAAGGGGATTGCA	3.77E-04	N	TetR-family transcriptional regulator
3548332	26	EC042_3327	*ygiB*	-88.5	CGGGTTATCTGCCGCA	1.34E-02	N	Putative lipoprotein
4219192	26	EC042_3978	*yicG*	-156.5	TATGCATTTTCTCAGA	8.93E-03	N	Putative membrane protein
2315229	25	EC042_2225	*yeeO*	-68.5	TTCGAGTCCAGTCAGA	1.25E-03	N	Putative membrane protein
2349836	25	*sbcB*	*dacD/sbcB*	-72.5	TGTGACTACTATCTCA	8.56E-05	N	Penicillin-binding protein 6B/ exodeoxyribonuclease I
4968382	25	*frdA*	*frdA*	-76.5	TGCGAACGCTATTCCA	3.49E-03	N	Fumarate reductase flavoprotein subunit
2551890	24	EC042_2422	*intA*	-239.5	GGTGTCGGGGGTCGGA	3.12E-03	N	Integrase
3276979	24	EC042_3075	Uncommon	-236.5	GGCGAAGGGAATCGAA	1.69E-03	-	Conserved hypothetical protein

3363224	24	EC042_3148	*yqgD*	133.5	TGAGACACGATTCAAA	9.37E-04	N	Putative membrane protein
4697783	24	*iclR*	*metH*	-96.5	TGTTGAACAAATCTCA	4.58E-03	N	Methionine synthase (5-methyltetrahydrofolate-homocysteine methyltransferase)
2733499	23	EC042_2583	*yfcZ/fadL*	-113.5	AGTGACCGAAATCACA	5.71E-06	Y	Conserved hypothetical protein/ Long-chain fatty acid transport protein
4243275	23	EC042_3998	Uncommon	-20.5	CGCAATCAACGCCACA	8.78E-04	-	Putative phage immunity repressor protein
2273560	22	EC042_2207	In K-12	-395.5	TTCGAGTCCAGTCAGA	1.25E-03	-	Integrase
2685148	22	EC042_2536	*yfbV*	-156.5	TTGGCTGAAAATTACG	1.84E-02	N	Putative membrane protein
4301258	22	EC042_4041	*yidE*	-71.5	TTTGCCTTATAGCGCA	4.65E-04	N	Putative transporter
5010825	22	*ulaA*	*ulaA*	-113.5	TGCGGGTCGCGTCACA	3.81E-05	Y	PTS ascorbate transporter subunit IIC
4236685	21	EC042_3992	In K-12	-169.5	TGTGATCTTCCGCCAA	5.71E-04	-	Tyrosine-type recombinase/integrase
2132403	20	*flhD*	*cutC*	-270.5	TGTGATGCAGATCACA	7.72E-09	N	Copper homeostasis protein
3590224	20	*air*	*air*	-93.5	TTTGATTTAGATCGCA	8.25E-06	Y	Aerotaxis receptor protein
3690953	20	EC042_3465	Uncommon	-68.5	TTTGGTACCGAGGACG	8.93E-03	-	Hypothetical protein
3723245	20	*gltB*	*gltB*	-188.5	TTTGCGCTAAAGCACA	1.14E-04	Y	Glutamate synthase [NADPH] large subunit
3948285	20	EC042_3720	*panM*	-204.5	CGTTGTCAGCATAAAA	1.01E-02	N	Putative acetyltransferase
965159	19	EC042_0908	*opgE*	-28.5	TGCGCGGTTTGTCATA	1.13E-03	N	Putative membrane protein
2158975	18	EC042_2067	*ftnB*	-171.5	TGTGATGTAAATCACA	5.37E-08	N	Ferritin-like protein 2
3512048	18	EC042_3292	*gpr*	-76.5	TGTGAGCCAGACTACG	3.15E-04	N	Putative aldo/keto reductase
1926713	17	*infC*	*lpp*	-853.5	CGTTAACTTCATCGCG	5.90E-04	N	Major outer membrane lipoprotein
3640881	17	*garK*	*garK*	1483.5	CGCTCACTGGCTCAAG	7.70E-03	N	Glycerate kinase 2
3858617	17	EC042_3644	In K-12	-83.5	GGCGAGGCGCTTCACA	9.67E-05	-	Hypothetical protein
4431631	17	*rho*	*rho*	-210.5	TGTAATTTCCAACGCT	8.30E-03	N	Transcription termination factor
336180	16	EC042_0305	Uncommon	938.5	TCCACCGTGATTCACG	1.14E-02	-	Conserved hypothetical protein
3327528	16	*gcvT*	*gcvT*	-87.5	CCCGGTCCCCAACGCA	7.32E-03	Y	Aminomethyltransferase (glycine cleavage system protein
4182076	16	*rfaK*	*rfaK*	658.5	TGTGAGCAACGGCCAA	6.53E-04	N	Lipopolysaccharide N-acetylglucosaminyltransferase

4318893	16	*dnaA*	*dnaA*	-189.5	TCTTCTGTTTCTCACA	1.69E-03	N	Chromosomal replication initiator protein DnaA
3846061	15	*frlA*	*frlA*	-115.5	TGTGATCTTCCTCCAC	1.37E-03	Y	Fructoselysine (psicoselysine) transporter
2313387	14	EC042_2224	*yeeN*	1185.5	TCTGACTGGACTCGAA	1.25E-03	N	Conserved hypothetical protein
3996354	14	EC042_3771	*ybhG*	-224.5	TGTGATCTATAAAACT	1.59E-03	N	HlyD family secretion protein
4944103	14	EC042_4606	Uncommon	-645.5	GGCGGCGTTCAGCCCA	4.71E-03	-	Putative helicase (pseudogene)
1302952	13	*dadA*	*minC*	-105.5	TGTGAGCCAGCTCACC	7.43E-06	N	Septum site determining protein MinC
4192231	13	*rpmB*	*rpmB*	-173.5	TGTGCTCAAGTCCCGA	2.79E-03	N	50S ribosomal subunit protein L28
4596821	13	*cytR*	*cytR*	-136.5	TTCGATCCGCCTCGCA	5.87E-05	Y	DNA-binding transcriptional regulator
5093367	13	EC042_4746	Uncommon	-657.5	GGAGAGCGACTCTGCA	3.32E-02	-	Conserved hypothetical protein
27718	12	*ribF*	*rpsT*	-90.5	TGTGCAAATAAGCGCC	2.71E-03	N	30S ribosomal protein S20
784980	12	EC042_0709	*In K-12*	-85.5	AGCGAGACTTTTCTCA	4.96E-03	Y	Putative exported protein
1127654	12	EC042_1056	In K-12	-273.5	GGTGCCTTCAACCGCT	5.66E-03	-	Conserved hypothetical protein
4956885	12	EC042_4617	*groS*	-136.5	TTTTGTGCTGATCAGA	3.79E-03	N	10 kDa chaperonin
4354065	11	EC042_4087A	*In K-12*	351.5	TTTGCTCAGGCTCTCC	3.39E-03	-	Putative plasmid-related protein
4852738	11	EC042_4524	Uncommon	-81.5	TCCTATAATGATCAAA	4.71E-03	-	Putative type VI secretion protein
3337434	10	*sbm*	*scpA*	-136.5	CTTGAATCACATCACA	6.13E-05	N	Methylmalonyl-CoA mutase
4999291	10	*EC042_4657*	*In K-12*	37.5	AGTAACGATAATCTCA	7.70E-03	-	Putative D-galactarate dehydratase/altronate hydrolase
407967	9	*EC042_0363*	Uncommon	179.5	TATGAAAAAAATCATA	5.37E-03	-	Hypothetical protein
3187106	9	*syd*	*syd*	219.5	TGTGGGTTGTATCACA	1.57E-05	N	Putative SecY-interacting protein
3883094	9	*greB*	*greB*	102.5	GGTGACCTGGGCCGCA	5.40E-05	N	Transcription elongation factor GreB
4177213	9	*rfaD*	*htrL*	-115.5	TTTGTTGCAATTAGCA	7.51E-03	N	Putative lipopolysaccharide biosynthesis protein
927587	8	EC042_0880	*moaA*	-45.5	TGTAGTCGGCGTCACA	1.28E-04	N	Molybdenum cofactor biosynthesis protein A
1149859	8	*putP*	*cspG*	-196.5	TGTGAGAGAGTGCAAC	1.10E-03	N	Old shock-like protein
4946926	8	*insB*	*insA*	57.5	TTTGCCGTTACGCACC	2.28E-03	N	Transposase

1068899	7	*serC*	*focA*	-144.5	TGTGATGCAAGCCACA	3.11E-06	Y	Probable formate transporter 1
2525169	7	EC042_2399	*psuK*	-219.5	TGTGACGGGATGCACA	1.06E-05	N	Putative pseudouridine kinase
3419417	7	*mchS1*	Uncommon	905.5	GTGGAGTGACAGCACT	2.39E-02	-	Putative microcin esterase
4761274	7	EC042_4436	pseudo	-125.5	GGTGATGCATTTTGCA	1.85E-03	-	Putative type III effector protein (pentapeptide repeat protein) (pseudogene)
20866	6	*insB*	*insB*	140.5	GGTGACAGCAGGCTCA	2.08E-03	N	IS1 transposase B
516051	6	*cyoA*	*cyoA*	-234.5	TGCGAAATAAAACAAT	7.32E-03	Y	Cytochrome o ubiquinol oxidase subunit 2
2746629	6	EC042_2591	*insA*	-11.5	CGCGCAGACGATGACG	4.46E-03	N	Putative transposase (IS1)
2945729	6	EC042_2760	*glrK*	-163.5	TTGGACGGCAGGCACC	5.81E-03	N	Two-component system sensor kinase
128586	5	*pdhR*	*pdhR*	-128.5	TGTTAAAATGTGCACA	6.98E-04	Y	Pyruvate dehydrogenase complex repressor
4260185	5	EC042_4011	Uncommon	2631.5	GCGGACTCACTTCACC	1.65E-02	-	Transcriptional regulator
3565809	4	EC042_3343	In K-12	1818.5	TCCGGCGTTCATCAAC	6.28E-03	-	Putative membrane protein
4213344	4	EC042_3970	Uncommon	308.5	TTTGCTCTCATCCACA	3.15E-04	-	Putative prophage protein
4266116	4	EC042_4012	Uncommon	4608.5	CGCGACAAACGTCACG	4.95E-05	-	Putative invasion ‘air’
5141146	4	EC042_4800	In K-12	-169.5	TATGAATGAAAGAACA	5.81E-03	-	Conserved hypothetical protein
5240772	4	*arcA*	*arcA*	-88.5	CATGATCGGCGTAACA	1.17E-03	N	Aerobic respiration control protein
1235648	3	*ptsG*	*rluC*	-142.5	CGTGATAGCCGTCAAA	5.17E-05	N	Ribosomal large subunit pseudouridine synthase C
1601165	3	EC042_1553	*insA1*	-85.5	CACGATCCCGCTCGCA	6.75E-04	N	Transposase
2842820	3	*dapA*	*dapA*	+ 0.5	CGTGAACATGGGCCAT	1.16E-02	N	Dihydrodipicolinate synthase
4362407	3	EC042_4096	In K-12	279.5	TCTGCACAAGCTCGCG	6.75E-04	-	Putative acetyltransferase
5118152	2	EC042_4771	In K-12	105.5	CCTGCAGACAAGCAGA	1.03E-02	-	Glycosyl transferase
5133814	2	EC042_4789	In K-12	852.5	AGCGAACCACACTGCA	6.44E-03	-	Transposase
5089682	1	EC042_4744	Uncommon	-1205.5	TTTCATATAGGCCGCG	5.52E-03	-	Conserved hypothetical protein

Targets on plasmid pAA							
27988	2975	*pet*	Uncommon	-117.5	CGAGAGCATTGTCACA	2.63E-04	Serine protease autotransporter toxin Pet
62001	907	EC042_RS26580	*traS*	-247.5	TGTGACCATATTATCA	1.33E-03	Hypothetical protein
18257	387	*virK*	Uncommon	-235.5	TGTGGTGACTGGTACA	1.37E-03	Virulence protein
112767	321	*repA*	In K-12	-84.5	ACAGATCTTCGTCACA	1.25E-03	Replication protein A
108769	293	*repA*	In K-12	-84.5	TGTGACGAAGATCTGT	1.25E-03	Replication protein A
39765	81	*aafA*	Uncommon	159.5	CGTTGACAGGAGCGCA	2.41E-03	Aggregative adherence fimbria II major subunit AafA
94581	23	EC042_RS26810	In K-12	-26.5	GTTGACTGGAGCCGCA	2.63E-03	Hypothetical protein
50078	15	EC042_RS29140	pseudo	-93.5	TGTGAAATTAATCAAA	5.40E-05	IS66 family insertion sequence element accessory protein TnpB
3987	13	EC042_RS30500	pseudo	186.5	TGAGCCCGCCATCACC	5.15E-04	Transposase
86933	13	EC042_RS26755	*hok*	-115.5	TCTGCCACACGACACG	2.56E-03	Type I toxin-antitoxin system Hok family toxin

[a] Peak centre indicated by MACS2 (8) and annotated to EAEC 042 chromosome. Locations with more than one CRP site are highlighted in red.

[b] Score (int(-10*log10qvalue)) assigned by MACS2.

[c] Annotated gene to an identified CRP site.

[d] EAEC 042 homologues to *E. coli* K-12 genes. Genes underlined represent matching genes targeted by CRP and previously indicated by Grainger *et a l*(9) . “Uncommon” denotes genes not found in K-12. “In K-12" indicates genes that are present in *E. coli* K-12 but with unknown functions.

[e] Position of identified CRP site to nearest TSS (Translation Start Site).

[f] Matching sequence obtained from MEME SUITE (10).

[g] *p*-value for CRP motif matches from MEME SUITE (10).

[h] Reported to be regulated by CRP, as listed for *E. coli* K-12 in RegulonDB(11).

[i] Product of encoding gene (12).

Table S3: A comparison of CRP-dependent promoters in *E. coli* K-12 and *E. coli* 042

Promoter	Strain (gene name is provided if in 042)	Promoter sequence [a]	bp distance CRP to TSS [b]	bp spacing CRP site to -35 [b]	bp spacing CRP to 10[b]	bp spacing -35 to -10[b]	"+1" position (K-12) [a]	% Identity [c]
exuTp2	(K-12)<br>EC042_3387	ATATTCCCACATTTGTGATGGCTCTCACCTTTTAAAGTTGTATGACAAGTTATCTTTCTGCCGTCGCAAATCATAAGTCGA<br>ATATTCCCACATTTGTGATGGCTCTCACCTTTTAAAGTTGTATGACAAGTTATCTTTCTGCCGTCGCAAATCATAAGTCGA	-39.5	N/A	30	N/A	3245002	100
lacZp2<br>*lacZ*	(K-12)<br>EC042_0381	GCAACGCAATTAATGTGAGTTAGCTCACTCATTAGGCACCCCAGGCTTTACACTTTATGCTTCCGGCTCGTATGTTGTGTG<br>GCAACGCAATTAATGTGAGTCAGCTCACTCATTAGGCACCCCAGGCTTTACACTTTATGCGTCCGGCTCGTATGTTGTGTG	-39.5	N/A	30	N/A	366365	97.5
acnAp2	(K-12)<br>EC042_1401	TCTTTTATCAATTTGGGTTGTTATCAAATCGTTACGCGATGTTTGTGTTATCTTTAATATTCACCCTGAAGAGAATCAGGG<br>TCTTTTATCAATTTGGGTTGTTATCAAATCGTTACGCGATGTTTGTGTTATCTTTAATATTCACCCTGAAGAGAATCAGGG	-40.5	N/A	29	N/A	1335781	100
deoCp2	(K-12)<br>EC042_4878	GATTTCCTTAATTGTGATGTGTATCGAAGTGTGTTGCGGAGTAGATGTTAGAATACTAACAAACTCGCAAGGTGAATTTTA<br>GATTTCCTTAATTGTGATGTATATCGAAGTGTGTTGCGGAGTAGATGTTAGAATACTAACAAACTCGCAAGGTGAATTTTA	-40.5	N/A	29	N/A	4617278	98.8
focAp1	(K-12)<br>EC042_0994	AGCCAGGCGAGATATGATCTATATCAATTTCTCATCTATAATGCTTTGTTAGTATCTCGTCGCCGACTTAATAAAGAGAGA<br>AGCCAGGCGAGATATGATCTATATCAATTTCTCATCTATAATGCTTTGTTAGTATCTCGTCGCCGACTTAATAAAGAGAGA	-40.5	N/A	30	N/A	954493	100
fucPp	(K-12)<br>EC042_3000	CTAGCTAATAAGTGTGACCGCCGTCATATTACAGAGCGTTTTTTATTTGAAAATGAATCCATGAGTTCATTTCAGACAGGC<br>CCAGCTAATAAGTGTGACCGCCGTCATATTACAGAGCGTTTTTTATTTGAAAATGAATCCATGAGTTCATTTCTGACAGGC	-40.5	N/A	29	N/A	2934119	97.5
gatY	(K-12)<br>EC042_2328	ATTGTCGTTTTTGTGATCGTTATCTCGATATTTAAAAACAAATAATTTCATTATATTTTGAAATCGAAAACAAACGACAG<br>ATTGTCGTTTTTGTGATCGTTATCTCGACATTTAAAAACAAATCATTTCATTATGTTTTGAAATCGAAAATAAACGACAG	-40.5	N/A	30	N/A	2177234	95
glgSp1	(K-12)<br>EC042_3341	TTCACTAAAAAGTGTGATCGGGGACAATATATTTACGCACGTTATGTTTAAAGGCACTACACTGATTGGGGAATACTGAAA<br>TTCACTAAAAAGTGTGATCGGGGACAATATATTTACGCACGTTATGTTTAAAGGCACTACACTGATTGGGGAATACTGAAA	-40.5	N/A	29	N/A	3192012	100
glpA	(K-12)<br>EC042_2484	AATGTTCAAAATGACGCATGAAATCACGTTTCACTTTCGAATTATGAGCGAATATGCGCGAAATCAAACAATTCATGTTTT<br>AATGTTCAGAATGACGCATGAAATCACGTTTCACTTTCGAATTATGAGCGAATATGCGCGAAATCAAACAATTCATGTTTT	-40.5	N/A	30	N/A	2352583	98.8
grcAp1	(K-12)<br>EC042_2785	TGTGTGGTTTTTATTGATTTAAATCAAAGATTCAAGGGTGTTTGAGGAGTATATATACACTCAAGCAACAATGGTTTTACC<br>TATGTGTTTTTTATTGATTTAAATCAAAGAATCAAGGGTGATTGAGGAGTATATATACACTCAAGCAACAATGGTTTTACC	-40.5	N/A	30	N/A	2716523	95.1
manX	(K-12)<br>EC042_1982	GAAAGTTAAATTACGGATCTTCATCACATAAAATAATTTTTTCGATATCTAAAATAAATCGCGAAACGCAGGGGTTTTTGG<br>GAAAGTTAAATTACGGATCTTCATCACATAAAATAATTTTTTCGATATCTAAAATAAATCGCGAAACGCAGGGGTTTTTGG	-40.5	N/A	30	N/A	1901933	100
mhpRp1	(K-12)<br>EC042_0383	ACTCGGACAAAATGTCGTTGCGCGCACAGTACAGCGCAACTTATTTTGTTAAAAACATGTAAATGATTTTTTATTGTGCGC<br>ACTCGGACAAAATGCCGTTGCGCGCACAGTACAGCGCAACTTATTTTGTTAAAAACATGTAAATGATTTTTTATTGTGCGC	-40.5	N/A	30	N/A	368536	98.8
nupC	(K-12)<br>EC042_2611	ACGTCATTATAGTGTGTGTCAGATCTCGTTTTCCTTAACCATGTTACATAGAATGTGCACGGAAATTTAACCTGCCTCATA<br>ACGTCATTATAGTGTGTGTCAGATCTCGTTTTCCTTAACCATGTTACATAGAATGTGCACGGAAATTTAACCTGCCTCATA	-40.5	N/A	29	N/A	2513009	100
nupG	(K-12)<br>EC042_3171	TTGCAATTATTTGCCACAGGTAACAAAAAACCAGTCCGCGAAGTTGATAGAATCCCATCATCTCGCACGGTCAAATGTGC<br>TTGCAATTATTTGCCACAGGTAACAAAAAACCAGTCCGCGAAGTTGATAGAATCCCATCATCTCGCACGGTCAAATGTGC	-40.5	N/A	29	N/A	3105651	96.2
ptsG	(K-12)<br>EC042_1171	CTGAAGTTGAAACGTGATAGCCGTCAAACAAATTGGCACTGAATTATTTTACTCTGTGTAATAAATAAAGGGCGCTTAGAT<br>CTGAAGTTGAAACGTGATAGCCGTCAAACAAATCGGCACTGAATTATTTTACTCTGTGTAATAAATAAAGGGCGCTTAGAT	-40.5	N/A	30	N/A	1157766	98.8
tsx	(K-12)<br>EC042_0446	AATGATAGAACTGTGAAACGAAACATATTTTTGTGAGCAATGATTTTTATAATAGGCTCCTCTGTATACGAAATATTTAG<br>AATGATAGAACTGTGAAACGAAACATATTTTTGTGAGCAATGATTTTTATAATAGGCTCCTCTGTATACGAAATATTTAG	-40.5	N/A	29	N/A	432091	100
bglGp2	(K-12)	AAGTTAATAACTGCGAGCATGGTCATATTTTTATCAATAGCGCATTGCTATTTTCTCTGCACGCAATTAAATTAATTTCCG	-41.5	N/A	30	N/A	3906717	
caiF	(K-12)<br>EC042_0036	GATGACATAAGCAGGATTTAGCTCACACTTATCGACGGTGAAGTTGCATACTATCGATATATCCACAATTTTAATATGGCC<br>GATGACATAAGCAGGATTTAGCTCACACTTATCGACGGTGAAGTTGCATACTATCGATATATCCACAATTTTAATATGGCC	-41.5	N/A	30	N/A	34218	100
caiT	(K-12)<br>EC042_0042	CGAAACAAAAATGTGATACCAATCACAGAATACAGCTTATTGAATACCCATTATGAGTTAGCCATTAACGCGTCCACGAGG<br>CGAAACAAAAATGTGACACCAATCACAGAATACAGCTTATTGAATACCCATTATGAGTTAGCCATTAACGCGTCCACGAGG	-41.5	N/A	30	N/A	42037	98.8
galEp1	(K-12)<br>EC042_0779	GATTCCACTAATTTATTCCATGTCACACTTTTCGCATCTTTGTTATGCTATGGTTATTTCATACCATAAGCCTAATGGAGC<br>GATTCCACTAATTTATTCCATGTCACACTTTTCGCATCTTTGTTATGCTATGGTTATTTCATACCATAAGCCTAATGGAGC	-41.5	N/A	30	N/A	792081	100
glpT<br>*glpT*	(K-12)<br>EC042_2483	ATTTAATAATGTGTGCGGCAATTCACATTTAATTTATGAATGTTTTCTTAACATCGCGGCAACTCAAGAAACGGCAGGTTC<br>ATTTAATAATGTGTGCGGTAATTCACATTTAATTTATGAATGTTTTCTTAACATCGCGGCAACTCAAGAAACGGCAGGTTC	-41.5	N/A	30	N/A	2352451	98.8
gntP	(K-12)<br>EC042_4826	TGATCAGAGGATGTGACATTCATCGCAACAATGGTTGACCAATTTACATAACATATCGCCCAAAATAACAACGGTTCAACC<br>TGATCAGAGGATGTGACATTCATCGCAACAATGGTTGACCAATTTACATAACATGTCGCCCAAAATAACAACGGTTCAACC	-41.5	N/A	30	N/A	4551334	98.8
gntX	(K-12)<br>EC042_3674	CTAACCAAATGGCTTTATCAGGTGCCTGTTGCAGCACGGCTTCGGCCATATCAGCAAGTGACAGCGCACCAAATCCCCGGC<br>CTAACCAAATGGCTTTATCAGGTGCCTGTCGCAGCACGGCTTCGGCCATATCAGCAAGCGACAGCGCACCAAATCCCCGGC	-41.5	N/A	30	N/A	3544667	97.5
idnDp	(K-12)	CAATTTTCTGACGTGATCTTCATCACAAATAATGACAGTTAAAACCGCTTAAATGCTTCCGAGGTGTCTACCTGACCAGTG	-41.5	N/A	31	N/A	4494435	

malX	(K-12)<br>EC042_1789	TCGTTGCGTAATGTGATTTATGCCTCACTAAAATTTGATAAAACGTTTTATCTTCTCGCGCAATTTACTGAATCCAGATTG<br>TCGTTGCGTAATGTGATTTATGCCTCACTATAATTTGATAAAACGTTTTATCTTCTCGCGCAATTTACTGAATCCAGATTG	-41.5	N/A	30	N/A	1699313	98.8
melR	(K-12)<br>EC042_4484	AGGGTGAAAACCGTGCTCCCACTCGCAGTCATCCTCCCTCACTCCTGCCATAATTCTGATATTCCAGGAAAGAGAGCCATC<br>AGGGTGAAAACCGTGCTCCCACTCGCAGTCATCCTCCCTCACTCCTGCCATAATTCTGATATTCCAGGAAAGAGAGCCATC	-41.5	N/A	30	N/A	4341650	100
mglB	(K-12)<br>EC042_2383	CGCTTTCAATCTGTGAGTGATTTCACAGTATCTTAACAATGTGATAGCTATGATTGCACCGTTTTAACGTTGTAACCCGTA<br>CGCTTTCAATCTGTGAGTGATTTCACAGTATCTTAACAATGTGATAGCTATGATTGCACCGTTTTAACGTTGTAACCCGTA	-41.5	N/A	30	N/A	2240566	100
nirB	(K-12)<br>EC042_3627	ATAAAGGTGAATTTGATTTACATCAATAAGCGGGGTTGCTGAATCGTTAAGGTAGGCGGTAATAGAAAAGAAATCGAGGCA<br>ATAAAGGTGAATTTGATTTACATCAATAAGCGGGGTTGCTGAATCGTTAAGGTAGGCGGTAATAGAAAAGAAATCGAGGCA	-41.5	N/A	29	N/A	3493987	100
pkap	(K-12)<br>EC042_2790	AAAACGCAACATCTGGGTAGCATCACAGCAGAACAGTTAGAAAGCGTTTAAAATCATTCGGTCACTTCTGCGGGAGACCGG<br>AAAACGCAACATCTGGGTAGCATCACAGCAGAACAGTTAGAAAGCGTTTAAAATCATTCGGTCACTTCTGCGGGAGACCGG	-41.5	N/A	30	N/A	2719931	
rpoHp5<br>*rpoH*	(K-12)<br>EC042_3722	GCATTGAACTTGTGGATAAAATCACGGTCTGATAAAACAGTGAATGATAACCTCGTTGCTCTTAAGCTCTGGCACAGTTG<br>GCATTGAACTTGTGGATAAAATCACGGTCTGATAAAACAGTGAATGATAACCTCGTTGCTCTTAAGCTCTGGCACAGTTG	-41.5	N/A	30	N/A	3600849	100
srlA	(K-12)<br>EC042_2895	ATCTTTCATTTTGCGATCAAAATAACACTTTTAAATCTTTCAATCTGATTAGATTAGGTTGCCGTTTGGTAATAAAACAAT<br>ATCTTTCATTTTGCGATCAAAATAACACTTTTAAATCTTTCAATCTGATTAGATTAGGTTGCCGTTTGGTAATAAAACAAT	-41.5	N/A	31	N/A	2825791	100
udp	(K-12)<br>EC042_4211	ATTTGCGTCATGGTGATGAGTATCACGAAAAAATGTTAAACCCTTCGGTAAAGTGTCTTTTTGCTTCTTCTGACTAAACCG<br>ATTTTCGTCATGGTGATGGGTATCACGAAAAAATGTTAAACCCTTCGGTAAAGTGTCTTTTTGCTTCTTCTGACTAAACCG	-41.5	N/A	30	97.5	4016391	97.5
ychH	(K-12)<br>EC042_1262	AGGGTTGTAATTGTGATCACGCCCGCACATAACCCACTGGGTGTTGTCTATACTTTACACATAAGGAAGAGGGGTATTCCC<br>AGGGTTGTAATTGTGATCACGCCCGCACATAACCCACAGGGTGTTGTCTATACTTTACACATAAGGAAGAGGGGTATTCCC	-41.5	N/A	30	N/A	1258738	98.8
ykgR	(K-12)	CTATTCAAATTTGTGATACATGTCAAAAAATTGGTTGACCAAACTCGTTATTTATATAAGGGCACTTACGAAGTGCACTCT	-41.5	N/A	30	N/A	313285	
araF	(K-12)<br>EC042_2064	AATTCTGCGATGTGATATTGCTCTCCTATGGAGAATTAATTTCTCGCTAAAACTATGTCAACACAGTCACTTATCTTTTAG<br>AATTCTGCGATGTGATATTGCTCTCCTATGGAGAATTAATTTCTCGCTAAAACTATGTCAACACGGTCACTTATCTTTTAG	-42.5	N/A	30	N/A	1986238	98.8
cdd	(K-12)<br>EC042_2375	GCATAATTAATGAGATTCAGATCACATATAAAGCCACAACGGGTTCGTAAACTGTTATCCCATTACATGATTATGAGGCAA<br>GCATAATTAATGAGATTCAGATCACATATAAAGCCATAACGGGTTCGTAAACTGTTATCCCATTACATGATTATGAGGCAA	-42.5	N/A	30	N/A	2231819	98.8
csgDp1	(K-12)<br>EC042_1105	AGTTACATTTAGTTACATGTTTAACACTTGATTTAAGATTTGTAATGGCTAGATTGAAATCAGATGTAATCCATTAGTTTT<br>AGTTACATTTAGTTACATGTTTAACACTTGATTTAAGATTTGTAATGGCTAGATTGAAATCAGATGTAATCCATTAGTTTT	-42.5	N/A	32	N/A	1103344	100
csiE	(K-12)<br>EC042_2739	CTTGCCAACATTTCTGATGATTAGCATTCCCTTCGCCATTTCCTTGAGCAAACTTTAGCTATTCTTATCAATTATGCTTAT<br>CTTGCCAACATTTCTGATGATTAGCATTCCCTTCGCCATTTCCCTGAGCAAACTTTAGCTATTCTTATCAATTATGCTTAT	-42.5	N/A	31	N/A	2665401	98.8
cyaR	(K-12)	TGGAAAATTCTTAGAAACCGATCACATACAGCTGCATTTATTAAGGTTATCATCCGTTTCGCTGAAAAACATAACCCATAA	-42.5	N/A	30	N/A	2167114	
fepA	(K-12)<br>EC042_0621	CCATGTTTACTGTGCAATTTTTCATTGATTGCAGAAATATATTGATAATATTATTGATAACTATTTGCATTTGCAATAGCG<br>CCATGTTTACTGTGCAATTTTTCATTGATTGCAGAAATATATTGATAATATTATTGATAACTATTTGCATTTGCAATAGCG	-42.5	N/A	31	N/A	612667	100
galP	(K-12)<br>EC042_3150	ATTACACTGATGTGATTTGCTTCACATCTTTTTACGTCGTACTCACCTATCTTAATTCACAATAAAAAAATAACCATATTGG<br>ATTACACTGATGTGATTTGCTTCACATCTTTTTACGTCGTACTCACCTATCTTAATTCACAATAAAAAAATAACCATATTGG	-42.5	N/A	30	N/A	3088255	100
galS	(K-12)<br>EC042_2384	TTTCCATTGCTGTGACTCGATTCACGAAGTCCTGTATTCAGTGCTGACAAAATAGCCGCCAGCAAGCAGTCATTTACTGCA<br>TTTCCATTGCTGTGACTCGATTCACGAAGTCCTGTATTCAGTGCTGACAAAATAGCCGCCAGCAAGCAGTCATTTACTGCA	-42.5	N/A	30	N/A	2241710	100
ompW	(K-12)<br>EC042_1310	TCATATGTAATGTGATCTATGTAGGATCATTTGTTACTCCAATGTAGGTATATTCGTCACGTTTTTATAACCATAACGACG<br>TCATATGTAATGTGATCTATGTAGGATCATTTGTTACTCCAATGTAGGTATATTCGTCACGTTTTTATAACCATAACGACG	-42.5	N/A	31	N/A	1313991	100
ppiAp2	(K-12)<br>EC042_3625	CATTTTAAGAGGTGATTTTGATCACGGAATAAAAAGTGATCGTCAGGTTACATATATTTCAGATACGTAAAATTAGGTAAA<br>CAATATTTAAAGTGATTCTCATCACGAAATATACGTGATCATCAGGTTACATATATTTCAAGTGCGTAAAATTAGGTAAA	-42.5	N/A	31	N/A	3492395	80.2
ptsHp1	(K-12)<br>EC042_2624	GAATCGATTTTATGATTTGGTTCAATTCTTCCTTTAGCGGCATAATGTTTAATGACGTACGAAACGTCAGCGGTCAACACC<br>GAATCGATTTTATGATTTGGTTCAATTCTTCCTTTAGCGGCATAATGTTTAATGACGTACGAAACGTCAGCGGTCAACACC	-42.5	N/A	32	N/A	2533608	100
yeiW	(K-12)	CTTTCGGGCAAGTGGTATTCGCACTTTTGCTGGTGCAAAAAAGGTGGTACTGTGCGCGCTCATCAATCCGGTGGTTAACCT	-42.5	N/A	30	N/A	2265330	
yhfA	(K-12)<br>EC042_3618	GCACGGTAATGTGACGTCCTTTGCATACATGCAGTACATCAATGTATTACTGTAGCATCCTGACTGTTTTAGCATAGCTTT<br>GCACGGTAATGTGACGTCCTTTGCATACATGCAGTACATCAATGTATTACTGTAGCATCCTGACTGTTTTAGCATAGCTTT	-42.5	N/A	30	N/A	3485951	100
cpdB	(K-12)<br>EC042_4694	TGCGCCAACTGTGATAGTGTCATCATTTTCAAAGCGTAAAATTGTGGCATTCTTCACTGTTCTATAAGTAAGACGTTTATT<br>TGCGCCAACTGTGATAGTGTCATCATTTTCAAAGCGTAAAATTGTGGCATTCTTCACTGTTCTATAAGTAAGACGTTTATT	-43.5	N/A	31	N/A	4436629	100
dadAp1<br>*dadA*	(K-12)<br>EC042_1238	TCAGGGAGATGTGAGCCAGCTCACCATAAAAAAGCCGCATGTTGAATAATATTTTCAACTGAGTTATCAAGATGTGATTAG<br>TCAGGGAGATGTGAGCCAGCTCACCATAAAAAAGCCGTATGTTGAATAATATTTTCAACTGAGTTATCAAGATGTAATTAG	-43.5	N/A	30	N/A	1237509	97.5
malI	(K-12)<br>EC042_1788	AAATTTTAGTGAGGCATAAATCACATTACGCAACGATAATAGCGGGTATAAGATAAATAAAAGGTAAAACGTTTTATCTGT<br>AAATTATAGTGAGGCATAAATCACATTACGCAACGATATTAGCGGGTATAAGATAAATAAAAGGTAAAACGTTTTATCTGT	-43.5	N/A	30	N/A	1699228	97.5
tdcA	(K-12)<br>EC042_3410	AAGTTAATTTGTGAGTGGTCGCACATATCCTGTTCATTTCATTTTGATACACTTCATGCCGTCAATGAGGTAATTAACGTA<br>AAGTTAATTTGTGAGTGGTCGCACATATCCTGTTCATTTCATTTTGATACACTTCATGCCGTCAATGAGGTAATTAACGTA	-43.5	N/A	31	N/A	3267093	100
yeiP	(K-12)<br>EC042_2404	ACCAGCAAAAGTGCGAATACCACTTGCCCGAAAGGCCCGTCGCGAGTACTTTGTCGCGATATTTTTGACATTTTCGACTAC<br>ACCAGCAAAAGTGCGAATACCACTTGCCCGAAAGGCCCGTCGCGAGTACTTTGTCGCGATATTTTTGACATTTTCGACTAC	-44.5	N/A	31	N/A	2265417	100
aldA	(K-12)<br>EC042_1546	TTACACTTGTTTTTATGAAGCCCTTCACAGAATTGTCCTTTCACGATTCCGTCTCTCTGATGATTGATGTTAATTAACAATGTATTCACCGAAAACAAACA<br>TTACACTTGTTTTTATGAAGCCCTTCACAGAATTGTCCTTTCACGATGCCGTCTCTATGATGATTGATGTTAATTAACAATGCATTTACCAATAACAAACA	-59.5	26	50	18	1488190	94.1

crp	(K-12)<br>EC042_3620	CGCGCAACGGAAGGCGACCTGGGTCATGCTGAAGCGAGACACCAGGAGACACAAAGCGAAAGCTATGCTAAAACAGTCAGGATGCTACAGTAATACATTGA<br>CGCGCAACGGAAGGCGACCTGGGTCATGCTGAAGCGAGACACCAGGAGACACAAAGCGAAAGCTATGCTAAAACAGTCAGGATGCTACAGTAATACATTGA	-59.5	25	48	17	3485953	100
dadAp2	(K-12)	AGAGTCAGGGAGATGTGAGCCAGCTCACCATAAAAAAGCCGCATGTTGAATAATATTTTCAACTGAGTTATCAAGATGTGATTAGATTATTATTCTTTTAC	-59.5	25	48	17	1237525	
fadD	(K-12)<br>EC042_1970	GATAAAAATAAATAGTGACGCGCTTCGCAACCTTTTCGTTGGGTAATTATCAAGCTGGTATGATGAGTTAATATTATGTTAACGGCATGTATATCATTTGG<br>GATAAAAATAAATAGTGACGCGCTTCGCAACCTTTTCGTTGGGTAATTATCAAGCTGGTATGATGAGTTAATATTATGTTAACGGCATGTATATCATTTGG	-59.5	26	48	16	1889807	100
tnaC	(K-12)	CTCCCCGAACGATTGTGATTCGATTCACATTTAAACAATTTCAGAATAGACAAAAACTCTGAGTGTAATAATGTAGCCTCGTGTCTTGCGAGGATAAGTGC	-59.5	26	48	16	3888411	
aldB	(K-12)<br>EC042_3898	CTTGCCCGTAAATTCGTGATAGCTGTCGTAAAGCTGTTACCGACTGGCGAAGATTTCGCCAGTCACGTCTACCCTTGTTATACCTCACACCGCAAGGAGACG<br>CTTGCCCGTAAATTCGTGATAGCCGTCGTAAAGCTGTTACCGACTGGCGAAGATTTCGCCAGTCACGTCTACCCTTGTTATACCTCACACCGCAAGGAGACG	-60.5	26	49	17	3756535	99
glpF	(K-12)<br>EC042_4301	ATAACGCTCATTTTATGACGAGGCACACACATTTTAAGTTCGATATTTCTCGTTTTTGCTCGTTAACGATAAGTTTACAGCATGCCTACAAGCATCGTGGAG<br>ATAACGCTCATTTTATGACGAAGCACACACATTTTAAGTTCGATATTTCTCGTTTTTGCTCGTTAACGATAAGTTTACAGCATGCCTACAAGCATCGTGGAG	-60.5	26	49	17	4118161	99
kbaZ	(K-12)<br>EC042_3423	ATTATGTTTCTTTTGTGAATCAGATCAGAAAACCATTATCTTTCGTTTTATTTTTATCTCACCATGACGCAGTATCAACTGAAACAAAACGAAAGATTAATA<br>ATTATGTTTCTTTTGTGAATCAGATCAGAAAACTATTATCTTTCGTTTTATTTTTATCTCACCATGGCGCAGTATCAACTGAAACAAAACGAAAGATTAATA	-60.5	26	49	17	3278866	98
lsrR	(K-12)<br>EC042_0152	CGTAGAGTCAAACTGTGGTTGCCATCACAGATATAAATGAGCAAGAACTGAACAATTGCATTAAAGATTTAAATATGTTCAAAGTGAAGAATGAATTATGAC<br>CGTAGAGTCAAACTGTGGTTGCTATCACAGATATAAATGCGCAAGAACTGAACAATTGCATTAAAGATTTAAATATGTTCAAAGTGAAGAATGAATTATGAC	-60.5	27	49	16	1601257	98
mdhp1	(K-12)<br>EC042_3521	ATAAACGCTAAACTTGCGTGACTACACATTCTTGAGATGTGGTCATTGTAAACGGCAATTTTGTGGATTAAGGTCGCGGCAGCGGAGCAACATATCTTAGTT<br>ATAAACGCTAAACTTGCGTGACTACACATTCTTGAGATGTGGTCATTGTAAACGGCAATTTTGTGGATTAAGGTCGCGGCAGCGGAGCAACATATCTTAGTT	-60.5	25	48	17	3384313	100
metK	(K-12)<br>EC042_3149	GTTTTTAACAGAATGAGACACGATTCAAAAAAAAGTGGAAATAGGGTGAAGAATTGACCTAAAATAGCCATCCAGATGTTAATCCATCCATACCGATTAACA<br>GTTTTTAACAGAATGAGACACGATTCAAAAAAAAGTGGAAATAGGGTGAAGAATTGACCTAAAATAGCCATCCAGATGTTAATCCATCCATACCGATTAACA	-60.5	26	48	16	3086577	100
nanA	(K-12)<br>EC042_3509	ATAATGCCACTTTAGTGAAGCAGATCGCATTATAAGCTTTCTGTATGGGGTGTTGCTTAATTGATCTGGTATAACAGGTATAAAGGTATATCGTTTATCAGA<br>ATAATGCCACTTTAGTGAAGCAGATCGCATTATAAGCTTTCTGTATGGGGTGTTGCTTAATTGATCTGGTATAACAGGTATAAAGGTATATCGTTTATCAGA	-60.5	24	49	19	3373620	100
ppiAp3	(K-12)	TTTTAGGTGATTTTGTGATCTGTTTAAATGTTTTATTGCAATCGGTTGCTAAATTGCATTTTAAGAGGTGATTTTGATCACGGAATAAAAAGTGATCGTCAG	-60.5	25	49	18	3492430	
ptsHp5	(K-12)	TTAGGTGCTTTTTTGTGGCCTGCTTCAAACTTTCGCCCCTCCTGGCATTGATTCAGCCTGTCGGAACTGGTATTTAACCAGACTAATTATTTTGATGCGCGA	-60.5	27	50	17	2533498	
relA	(K-12)<br>EC042_2981	GATCGCGAAAAACTGGAACGCTTTTCGCATTCTGAAGGCCTGGATCTGTATCTCGCCCCCGATAGTGAGATACTCGAAACCGTCTCTGGTGAGATGCCCTGG<br>GATCGCGAAAAACTGGAACGCTTTTCGCATTCTGAAGGCCTGGATCTGTATCTCGCCCCCGATAGTGAGATACTCGAAACCGTCTCTGGTGAGATGCCCTGG	-60.5	25	50	19	2914277	100
treB	(K-12)<br>EC042_4718	CTGAACGATAAATTGTGATCTTCGCTGCGTTTCGGGAACGTTCCCGTTTTTAAATTTTTCCGCGCAATATATTCTGCAGCCAACCAAAAATGTCATCTGCCA<br>CTGAACGATAAATTGTGATCTTCGCTGCGTTTCGGGAACGTTCCCGTTTTTAAATTTTTCCGCGCAATATATTCTGCAGCCAACCAAAAATGTCATCTGCCA	-60.5<br>**(-61.5)**	26<br>**(27)**	49<br>**(50)**	17	4466210<br>**(+1)**	99
ulaA	(K-12)<br>EC042_4674	ATGTGGAATTATTTGCGGGTCGCGTCACATTTAATCATAAATAATCTTGTTGTGATTACTTTTGAAAATTAGAGTGAGTGCACAACATTCCGGGTGTGTGGA<br>ATGTGGAATTATTTGCGGGTCGCGTCACATTTAATCATAAATAATCTTGTTGTGATTACTTTTGAAAATTAGAGTGAGTGCACAACATTCCGGGTGTGTGGA	-60.5	26	49	17	4419927	100
bglGp	(K-12)	CAAAGTTAATAACTGCGAGCATGGTCATATTTTTATCAATAGCGCATTGCTATTTTCTCTGCACGCAATTAAATTAATTTCCGAACCTGGATGTTCGTTATAA	-61.5	26	49	17	3906697	
cspEp2	(K-12)<br>EC042_0659	ACATCAAAAATAACGCGACTTTTATCACTTTTTAGTAAAGTTACACTGGACAAAGCGTACCACAATTGGTGTACTGGTAACCGACACAGCATTTGTGTCTATT<br>ACATCAAAAATAACGCGACTTTTATCACTTTTTAGTAAAGTTGCACTGGACAAAGCGTACCACAATTGGTGTACTGGTAACCGACACAGCATTTGTGTCTATT	-61.5	26	49	17	657250	99
fadH	(K-12)<br>EC042_3374	CCGGGCGTATTCTTTTTGAATCCCATCACAAACCCCGCACTCCCCTTTTCCCTTTTCTCCGGCGACGGCTAAATTAGAACTCATCCGACCACATAACAATTAT<br>ACAGGCGTATTCTTTTTGAATCCCATCACAAACCCCACACTCCCCTTTTCCCTTTTCTCCGGCGACGGCTAAATTAGAACTCATCCGACCACATAACAATTAT	-61.5	26	49	17	3231624	97.1
lacZp1	(K-12)	GCAACGCAATTAATGTGAGTTAGCTCACTCATTAGGCACCCCAGGCTTTACACTTTATGCTTCCGGCTCGTATGTTGTGTGGAATTGTGAGCGGATAACAATT	-61.5	26	50	18	366343	
nagE	(K-12)<br>EC042_0707	AATAACGATATTTGGTGACAAAACTCACAAAAGACACGCGTTTAATTTGCGATACGAATTAAATTTTCACACACTCTGTAGCAGATGATCTAACAATCTGATT<br>GATAACGATATTTGGTGACAAAACTCACAAAAGACACGCGTTTAATTTGCGATACGAATTAAATTTTCACACACTCTGTAGCAGATGATCTAACAATCTGATT	-61.5	26	49	17	703840	99
nanC	(K-12)<br>EC042_4822	GATCGACATATTTTGTGACACGAATTGCAAATCTGGTTTTGTTGTATGGATTGCGTGATTTTTGATCTGGTATAACAGGTATAAAGGTGCACCAAGATAGTCA<br>GATCGACATATTTTGTGACACGAATTGCAAATCTGGTTTTGTTGTATGGATTGCGTGATTTTTGATCTGGTATAACAGGTATAAAGGTGCACCGCGATAGACA	-61.5	26	50	18	4539928	97.1
secB	(K-12)<br>EC042_3919	TTCCCCAGATTTTTATTGACGCACAGCACATTGGCGGCTGTGATGACTTGTATGCATTGGATGCACGTGGTGGACTGGATCCCCTGCTGAAATAACGTGTGAA<br>TTCCCCAGATTTTTATTGACGCACAGCACATTGGCGGCTGTGATGACTTGTATGCATTGGATGCACGTGGTGGACTGGATCCCCTGCTGAAATAACGTGTGAA	-61.5	27	50	17	3784203	100
gadA	(K-12)<br>EC042_3812	CGTTTTTCTGCTTAGGATTTTGTTATTTAAATTAAGCCTGTAATGCCTTGCTTCCATTGCGGATAAATCCTACTTTTTTATTGCCTTCAAATAAATTTAAGGAG<br>CGTTTTTCTGCTTAGGATTTTGTTATTTAAATTAAGCCTGTAATGCCTTGCTTCCATTGCGGATAAATCCTACTTTTTTATTGCCTTCAAATAAATTTAAGGAG	-62.5	27	50	17	3667607	100
modAp	(K-12)<br>EC042_0782	TGCCGATATTTTTTCTTATCTACCTCACAAAGGTTAGCAATAACTGCTGGGAAAATTCCGAGTTAGTCGTTATATTGTCGCCTACATAACGTTACATTAAGGGG<br>TGCCGATATTTTTTCTCATCTACCTCACAAAGGTTAGCAATAACTGCTGGGAAAATTCTGAGTTAGCCGTTATATTGACGCCTACATAACGTTACATTAAGGGG	-62.5	27	50	17	795062	96.2
rbsD	(K-12)<br>EC042_4135	CCATGTAAAACGTTTCGAGGTTGATCACATTTCCGTAACGTCACGATGGTTTTCCCAACTCAGTCAGGATTAAACTGTGGGTCAGCGAAACGTTTCGCTGATGG<br>CCATGTAAAACGTTTCGAGGTTGATCACATTTCCGTAACGTCACGATGGTTTTCCCAACTCAGTCAGGATTAAACTGCGGGTCAGCGAAACGTTTCGCTGATGG	-62.5	29	50	15	3933322	99
rpoS	(K-12)<br>EC042_2935	GCAGAGCAAGGAGTTGTGATCAAGCCTGCACAAAATTCCACCGTTGCTGTTGCGTCGCAACCGACAATTACGTATTCTGAGTCTTCGGGTGAACAGAGTGCTAA<br>GCAGAGCAAGGAGTTGTGATCAAGCCTGCACAAAATTCCACCGTTGCTGTTGCGTCGCAACCGACAATTACGTATTCTGAGTCTTCGGGTGAACAGAGTGCTAA	-62.5	29	52	17	2868118	100
aaeX	(K-12)<br>EC042_3527	ACTCTGACTTAAAAGTGATTTAGATCACATAATATAGATAACAGCATAACAGTTACGCTAATATATTAAATATCAATCTACAGCAATGTTGCTCTCGCCCGGCTT<br>ACTCTGACTTAAAAGTGATTTAGATCACATAATATAGATAACAGCATAACAGTTACGCTAATATATTAAATATCAATCTACAGCAATGTTGCTCTCGCCCGGCTT	-63.5	32	50	12	3389430	100
dusB	(K-12)<br>EC042_3543	TTTAAATGCAATTCTTTGATCCATCTCAGAGGATTGGTCAAAGTTTGGCCTTTCATCTCGTGCAAAAAATGCGTAATATACGCCGCCTTGCAGTCACAGTATGGT<br>TTTAAATGCAATTCTTTGATCCATCTCAGAGGATTGGTCAAAGTTTGGCCTTTCATCTCGTGCAAAAAATGCGTAATATACGTTGCAGTCACAGTATGGT	-63.5	30	53	17	3410246	95.2
gadB	(K-12)	ATAAATAACATTAGGATTTTGTTATTTAAACACGAGTCCTTTGCACTTGCTTACTTTATCGATAAATCCTACTTTTTTAATGCGATCCAATCATTTTAAGGAG	-63.5	32	51	13	1572072	
glpD	(K-12)<br>EC042_3686	ATAAACGCCATAATGTTATACATATCACTCTAAAATGTTTTTTCAATGTTACCTAAAGCGCGATTCTTTGCTAATATGTTCGATAACGAACATTTATGAGCTTTA<br>ATAAACGCCATAATGTTATACATATCACTCTAAAATGTTTTTTCAATGTTACCTAAAGCGCGATTCTTTGCTAATATGTTCGATAACGAACATTTGTGAGCTTTA	-63.5	32	51	13	3561971	99

ivbL	(K-12)<br>EC042_4026A	ATTCAGTACAAAACGTGATCAACCCCTCAATTTTCCCTTGCTGAAAAATTTTCCATTGTCTCCCCTGTAAAGCTGTGCTTGTATAAATATTGTTAAACACAAAAC<br>GTACAGTACAAAACGTGATCAACCCCTCAATTTTCCCTTGCTGAAAAATTTTCCATTGTCTCCCCTGTAAAGCTGTGCTTGTATAAATATTGTTAAACACAAAAC	-63.5	30	47	11	3853023	98.1
ldtB	(K-12)<br>EC042_0908	CAAAGGCAAAAAATGTGATTTCGTACACATCTGATTTCACTGTGAGCTGGAATGAACTTATAATGCGCTTCCAATACTCTAATATTCTCAACCCAATGGCCTGCC<br>CAAAGGCAAAAAATGTGATTTCGTACACATCTGATTTCACTGTGAGCTGGAATGAACTTATAATGCGCTTCCAATACTCTAATATTCTCAACCCAATGGCCTGCC	-63.5	32	51	13	855786	100
ptsHp4	(K-12)	TTAGGTGCTTTTTTGTGGCCTGCTTCAAACTTTCGCCCCTCCTGGCATTGATTCAGCCTGTCGGAACTGGTATTTAACCAGACTAATTATTTTGATGCGCGAAAT	-63.5	31	50	13	2533501	
cytR	(K-12)<br>EC042_4308	GCGGATCGAAAAATTCAATATTCATCACACTTTTCATGAAAATTCTGTAACCGTTTTCACGCGCTATCTGCTAAAAATGTTGCCGATGTGAAGTAAACATGGATGT<br>GCGGATCGAAAAATTCAATATTCATCACACTTTTCATGAAAATTCTGTAACCGTTTTCACGCGCTATCTGCTAAAAATGTTGCCGATGTGAAGTAAACATGGAAGT	-64.5	34	51	11	4124509	99.1
gltB	(K-12)<br>EC042_3502	TTTAATAAAGAATTTTGCGCTAAAGCACATTTCTGTACCAATAAGCTTGCCATTTGACCTGTATCAGCTTTCCCGATAAGTTGGAAATCCGCTGGAAGCTTTCTGGA<br>TTTAATAAAGAATTTTGCGCTAAAGCACATTTCTGTACCAATAAGCTTGCCATTTGACCTGTATCAGCTTTCCCGATAAGTTGGAAATCCGCTGGAAGCTTTCTGGA	-65.5	33	54	15	3354509	100
hpt	(K-12)<br>EC042_0124	AGGTTTTAACATGTGTGATCGTCATCACAATTCGAGCTTTATTAACAGATTCCGCGAATGAATAGTTTTACTGGTATACTGCGTGTCTTGCGCTTTGTTGCGGTGCC<br>AGGTTTTAACATGTGTGATCGTCATCACAATTCGAGCTTTATTAACAGATTCCGCGAATGAATAGTTTTACTGGTATACTGCGTGTCTTGCGCTTTGTTGCGGTGCC	-65.5	29	54	19	141360	100
sfsA	(K-12)<br>EC042_0146	ACTGGTCGTATGCGGTGACGGAGTTCACCCTTTACGCCTCCTCGTTTGCCCGTGGACGCACACGCTACACGCCGCTAAAACGCTGGGCGCTAACGCAATAACAAGGA<br>ACTGGTCGTATACGGTGACGGAGTTCACCCTTTACGCCTCCTCGTTTGCCCGTGGACGCACACGCTACACGCCGCTAAAACGCTGGGCGCTAACGCAATAACAAGGA	-65.5	32	55	17	161515	99.1
csiD	(K-12)<br>EC042_2856	ATGTTACTAATTTGTTGCTTTTGATCACAATAAGAAAACAATATGTCGCTTTTGTGCGCATTTTTCAGAAATGTAGATATTTTTAGATTATGGCTACGAAATGAGCATCG<br>ATGTTACCAATTTGTTGCTTTTGATCACAATAAGAAAACAATATGGCGCATTTGTGCGCATTTTTCAGAAATGTAGATATTTTTAGATTATGGCTACGAAATGAGCATCG	-68.5	31	57	20	2788927	97.3
gntK	(K-12)<br>EC042_3703	TTAAGCCCACAAATTTGAAGTAGCTCACACTTATACACTTAAGGCATGGATGGATATTGCTTCTGATATTGTCCGGCTGGACAATGTTACCGATAACAGTTACCCGTAAC<br>TTAAGCCCACAAATTTGAAGTAGCTCACACTTATACACTTAAGGCACGGATGGATATTGCTTCTGATATTGTCCGGCTGGACAATGTTACCGATAACAGTTACCCGTAAC	-68.5	36	59	17	3577645	99.1
prpR	(K-12)<br>EC042_0366	TAATCCGCAAATATGCGTTTCAGTTAACGTTTCAGGCAATGTTTCACCGCGTTTCATTGCAACAATTATGAAACAAGACTAAACCCAATATTCGGTTTCTTAACTTTGCG<br>TAATCCGCAAATATGCGTTTCAGTTAACGTTTCAGGCAATGTTTCACCGCGTTTCATTGCAACAATTATGAAACAAGACTAAACCCAATATTCAGTTTCTTAACTTTGCG	-68.5	36	59	17	348471	99.1
acs	(K-12)<br>EC042_4440	AATTCTACTTTTGCGTGATCTGTCGCCCAAATACTAAACAAAACTGCCAATACCCCTACATTTAACGCTTATGCCACATATTATTAACATCCTACAAGGAGAACAAAAGCA<br>AATTCTACTTTTGCGTGATCTGTCGCCCAAATACTAAACAAAACTGCCAATACCCCTACATTTAACGCTTATGCCACATATTATTAACATCCTACAAGGAGAACAAAAGCA	-69.5	36	58	16	4287391	100
fixA	(K-12)<br>EC042_0043	TGTTTTCAATATTGGTGAGGAACTTAACAATATTGAAAGTTGGATTTATCTGCGTGTGACATTTTCAATATTGGTGATTAAAGTTTTATTTCAAAATTAAAGGGCGTGATA<br>TGTTTTCAATATTGGTGAGGAACTTAACAATATTGAAAGTTGGATTTATCTGCGTGTGACATTTTCAATATTGGTGATTAAAGTTTTATTTCCAAATTAAAGGGCGTGATA	-69.5	35	58	17	42325	99.1
chiP	(K-12)<br>EC042_0709	CATCAAAATATGTGCGGTTGCTCATGTTCTTACATTCTGGTTACAGAAAGAGATTGATAATTCGCGTCGCGAAAAATAGTCTGTTCCTGTAGTCAGCGAGACTTTTCTC<br>CATCAAAATATGTGCGGTTGCTCATGTTCTTACATTCTGGTTACAGAAAGAGATTGATAATTCGCGTCGCGAAAAATAGTCTGTTCCTGTAGTCAGCGAGACTTTTCTC	-70.5	36	59	17	708236	100
epd	(K-12)<br>EC042_3136	AAGGGGATAAAAGTGTGATGTGAGTCAGATAAATGTCTTCTTCGGCTGGACAAACATTCCTTTTATTCCACGTTTCGCTTATCCTAGCTGAAGCGTTTCAGTCGATTAAATG<br>AAGGGGATAAAAGTGTGATGTGAGTCAGATAAATGTCTTCTTCGGCTGGACAAACATTCCTTTTATTCCACGTTTCGCTTATCCTAGCTGAAGCGTTTCAGTCGATTAAATG	-70.5	36	59	17	3073823	100
furpb	(K-12)<br>EC042_0711	TTGCCGTTGTAAATGTAAGCTGTGCCACGTTTTTATTAACAATATTTGCCAGGGACTTGTGGTTTTCATTTAGGCGTGGCAATTCTATAATGATACGCATTATCTCAAGAGC<br>TTGCCGTTGTAAATGTAAGCAGTGCCACGTTTTTATTAACAATATTTGCCAGGGACTTGTGGTTTTCATTTAGGCGTGGCAATTCTATAATGATACGCATTATCTCAAGAGC	-70.5	36	59	17	710723	99.1
idnK	(K-12)	ATGTTACGCATAACGTGATGTGCCTTGTAATTCTTATCAGTAGAAATAAAAAAACGTGAAATTATTATGCCGCCAGGCGTAGTATCGCAGCAGGTAAGATGATTCAGGAGAT	-70.5	36	59	17	4494597	
malT	(K-12)<br>EC042_3679	AAAGATTTGGAATTGTGACACAGTGCAAATTCAGACACATAAAAAAACGTCATCGCTTGCATTAGAAAGGTTTCTGGCCGACCTTATAACCATTAATTACGAAGCGCAAAAA<br>AAAGATTTGGAATTGTGACAGAGTGCAAATTCAGACACATAAAAAAACGTCATCGCTTGCATTAGAAAGGTTTCTGGCCGACCTTATAACCATTAATTACGAAGCGCAAAAA	-70.5	36	59	17	3553024	99.1
mlcp2	(K-12)<br>EC042_1742	AAAGCCCGAAAAATGTGCTGTTAATCACATGCCTAAGTAAAAATTTGACGACACGTATTGAAGTGCTTCACCATAGCCTACAGATTATTTCGGAGCGCGAAAATATAGGGAG<br>AAAGCCCGAAAAATGTGCTGTTAATCACATGCCTAAGTAAAAATTTGACGACACGTATTGAAGTGCTTCACCATAGCCTACAGATTATTTCGGAGCGCGAAAATATAGGGAG	-70.5	37	58	15	1668591	100
nagB	(K-12)<br>EC042_0706	AACGCGTGTCTTTTGTGAGTTTTGTCACCAAATATCGTTATTATCACTCCCTTTTACTGGCTAAACCAGAAAACTTATTTTATCATTCAAAAAATCAGGTCGGATTGACGCC<br>AACGCGTGTCTTTTGTGAGTTTTGTCACCAAATATCGTTATCATCACTCCCTTTTACTGGCTAAACCAGAAAACTTATTTTATCATTCAAAAAATCAGGTCGGATTGACGCC	-70.5	37	60	17	703708	99.1
ascF	(K-12)<br>EC042_2908	AAAATATTCAGGTGACCGGTTTCACAAATATAAAAAATGAACAATTCACTCTCTTGCTTATTTAGTGACAACTATTCATGATTTTGTGAAACCGGTTTCTTAATTCCGTT<br>AAAATATTCAGGTGACCGGTTTCACAAATATAAAAAATGAACAATTAACTCTCTTGCTTATTTAGTGACAACTATTCATGATTTTATGAAACCGGTTTCTTAATTCCGTT	-71.5	36	59	17	2839429	98.2
feaBp1	(K-12)	TATTGTCACGATTTGCGGAGCTTGTCACAGCTGACAAAGCGAATGTCACAGCGAAAAAAGTGACTTTTCTTGTCGCTGCGTACACTGAAATCACACTGGGTAAATAATAAGGA	-71.5	39	60	15	1447492	
flhD	(K-12)<br>EC042_2058	ATTGAGTGTTTTGTGTGATCTGCATCACGCATTATTGAAAATCGCAGCCCCCCTCCGTTGTATGTGCGTGTAGTGACGAGTACAGTTGCGTCGATTTAGGAAAAATCTTAGAT<br>ATTGAGTGTTTTGTGTGATCTGCATCACACATTATTGAAAATCGCAGCCCCCCTCCGTTGTATGTGCGTGTAGTGACGAGTACAGTTGCGTCGATTTAGGAAAAATCTTAGAT	-71.5	37	60	17	1978395	99.1
glnAp1	(K-12)<br>EC042_4243	TTGCAGAGTCCCTTTGTGATCGCTTTCACGGAGCATAAAAAGGGTTATCCAAAGGTCATTGCACCAACATGGTGCTTAATGTTTCCATTGAAGCACTATATTGGTGCAACATT<br>TTGCAGAGTCCCTTTGTGATCGCTTTCACGGAGCATAAAAAGGGTTATCCAAAGGTCATTGCACCAATATGGTGCTTAATGTTTCCATTGAAGCACTATATTGGTGCAACATT	-71.5	34	56	16	4058222	99.1
gntTp	(K-12)<br>EC042_3676	TTCCCCGCGATAATATGACCAACCTCTCATAATTTAAATTTACCCCGCTCTGGTGATTCTCAAACGCCAGATGTTACCCGTATCATTCACATGGGTACCAAACATACTCCTGA<br>TTCCCCGCGATAATATGACCAACCTCTCATAATTTAAATTTACCCCGCTCTGGTGATTCTCAAACGCCGGATGTTACCCGTATCATTCACGTGGGTACCAAACATACTCCTGA	-71.5	36	60	18	3546405	98.2
murQp1	(K-12)<br>EC042_2637	CCGCTTAAGTTCTTATGACGCTCTTCACACTCTGCGAGTGTAGACTCAATGATTCCTTTAAGCCTGTCAATTCGGGATATATGATTCCATACATCTGCAATTATTTGATCGTA<br>CCGCTTAAGTTCTTATGACGCTCTTCACACTCTGCGAGTGTAGACTCAATGATTCCTTTAAGTCTGTCAATTCGGGATATATGATTCCATACATCTGCAATTATTTGATCGTA	-71.5	37	59	16	2545733	99.1
dadAp3	(K-12)	AGAGTCAGGGAGATGTGAGCCAGCTCACCATAAAAAAGCCGCATGTTGAATAATATTTTCAACTGAGTTATCAAGATGTGATTAGATTATTATTCTTTTACTGTATCTACCGTT	-72.5	37	62	19	1237538	
rpoHp3	(K-12)	TCTTCCCGGTATTTCATCTCTATGTCACATTTTGTGCGTAATTTATTCACAAGCTTGCATTGAACTTGTGGATAAAATCACGGTCTGATAAAACAGTGAATGATAACCTCGTTGC	-73.5	39	62	17	3600870	
tsx	(K-12)	GAATGTGTGTAAACGTGAACGCAATCGATTACGTAAATGATAGAACTGTGAAACGAAACATATTTTTGTGAGCAATGATTTTTATAATAGGCTCCTCTGTATACGAAATATTTAG	-73.5	39	62	17	432091	
yaeQ	(K-12)<br>EC042_0189	ACTCTGTTTCGCAAGGTGACTATACCACACTCATTTCTGCAATATCAGCGCCGCAACTGCACGTATTCCGTTACAATGGCCTCCTGATTCGAAAGGAGTTTTCTTATGGCGCTTA<br>ACTCTGTTTCGCAAGGTGACTATACCACACTCATTTCTGCAATATCAGCGCCGCAACTGCACGTATTCCGTTACAATGGCCTCCTGATTCGAAAGGAGTTTTCTTATGGCGCTTA	-73.5	42	65	17	214280	100
ompR	(K-12)<br>EC042_3666	CGCACATTGGGTATAACGTGATCATATCAACAGAATCAATAATGTTTCGCCGAATAAATTGTATACTTAAGCTGCTGTTTAATATGCTTTGTAACAATTTAGGCTGAAATTCATAC<br>CGCACATTGGGTATAACGTGATCATATCAACAGAATCAATAATGTTTCGCCGAATAAATTGTATACTTAAGCTGCTGTTTAATATGCTTTGTAACAATTTAGGCTGAAATTCATAC	-74.5	38	62	18	3536685	100
malE	(K-12)<br>EC042_4403	TTACCGCCAATTCTGTAACAGAGATCACACAAAGCGACGGTGGGGCGTAGGGGCAAGGAGGATGGAAAGAGGTTGCCGTATAAAGAAACTAGAGTCCGTTTAGGTGTTTTCACGAGCA<br>TTACCGCCAATTCTGTAACAGAGATCACACAAAGCAACGGTGGGGCGTAGGGGCAAGGAGGATGGAAAGAGGTTGCTGTATAAAGAAACTAGAGTCCGTTTAGGTGTTTTCACGAGCA	-76.5	42	64	16	4246464	98.3

putP	(K-12)<br>EC042_1090	CAACCTGATGAAAAATAGTGTCGCTGAGCACTAAAATTTAATGTAAATGGTGTGTTAAATCGATTGTGAATAACCAGCGCTTCCGGCAGGATACGGTCGCCCTGGTAAAACATAAACT<br>CAACCTGATGAAAAATAGTGTCGCTGAGCACTAAAATTTAATGTAAATGGTGTGTTAAATCGATTGTGAATAACCAGCGCTTCCGGCAGGATACGGTCGCCCTGGTAAAACATAAACT	-76.5	43	66	17	1079210	100
furpa	(K-12)	TTGCCGTTGTAAATGTAAGCTGTGCCACGTTTTTATTAACAATATTTGCCAGGGACTTGTGGTTTTCATTTAGGCGTGGCAATTCTATAATGATACGCATTATCTCAAGAGCAAATTCT	-77.5	44	65	17	710716	
murQp2	(K-12)	CCGCTTAAGTTCTTATGACGCTCTTCACACTCTGCGAGTGTAGACTCAATGATTCCTTTAAGCCTGTCAATTCGGGATATATGATTCCATACATCTGCAATTATTTGATCGTAAATAGTAA	-79.5	43	67	18	2545741	
serA	(K-12)<br>EC042_3123	CCCCCGTTAAAAAAATTCTCTTCATTAAATTTGGTGACATGTGTCACGCTTTTACCAGGCAATTGTCGATTGCTCTAAATAAATCCTCTAAACCAGCATATTCATCCAAGAATTACCTTTG<br>CCCCCGTTAAAAAAATTCTCTTCATTAAATTTGGTGACATGTGTCACGCTTTTACCAGGCAATTGTCGATTGCTCTAAATAAATCCTCTAAACCAGCATATTCATCCAAGAATTACCT	-79.5	43	68	19	3058549	100
fucP	(K-12)	TTTCGATTATTAAAGTGATGGTAGTCACATAAAGTCACCTTCTAGCTAATAAGTGTGACCGCCGTCATATTACAGAGCGTTTTTTATTTGAAAATGAATCCATGAGTTCATTTCAGACAGGC	-80.5	43	69	20	2934119	
hofM	(K-12)<br>EC042_3656	GGCGCTGTAAATCTGCATCGGAATTTGCAGGCGAACATCTTTTAATGTCGCCACATCCGGCAGTTGTGGCTCGATGTAGCGGTATAGGCCATAAATCGAGCCTGCTCCCAGCAGAATGCAAC<br>GGCGCTGTAAATCTGCATCGGAATTTGCAGGCGAACATCTTTTAATGTCGCCACATCCGGCAGTTGTGGCTCGATGTAGCGGTATAGGCCATAAATCGAGCCTGCTCCCAGCAGAATG	-80.5	43	69	20	3522928	100
rpoHp4	(K-12)	TCTTCCCGGTATTTCATCTCTATGTCACATTTTGTGCGTAATTTATTCACAAGCTTGCATTGAACTTGTGGATAAAATCACGGTCTGATAAAACAGTGAATGATAACCTCGTTGCTCTTAAG	-80.5	45	68	17	3600863	
xylE	(K-12)	GATAATTATCACAATTAAGATCACAGAAAAGACATTACGTAAACGCATTGTAAAAAATGATAATTGCCTTAACTGCCTGACAATTCCAACATCAATGCACTGATAAAAGATCAGAATGG	-80.5	43	69	20	4242291	
melA	(K-12)<br>EC042_4485	TAAACTCAGATTTACTGCTGCTTCACGCAGGATCTGAGTTTATGGGAATGCTCAACCTGGAAGCCGGAGGTTTTCTGCAGATTCGCCTGCCATGATGAAGTTATTCAAGCAAGCCAGGAGATC<br>TAAACTCAGATTTATTGATGCTTCACGTAGGATCTGAGTTTATGGGAATGCTCAACCTGGAAGCCGGAGGTTTTCTGCAGATTCGCCTGCCATGATGAAGTTATTCAAGCAAGCCAGG	-81.5	49	70	15	4341887	97.6
pkap	(K-12)	GGCGAGCATTTTACCCGCTTTAAAACACGCTATCTGGCAGGAAAAACGCAACATCTGGGTAGCATCACAGCAGAACAGTTAGAAAGCGTTTAAAATCATTCGGTCACTTCTGCGGGAGACCGG	-81.5	47	70	17	2719931	
xylA	(K-12)<br>EC042_3871	TTCCATTTTATTTTGCGAGCGAGCGCACACTTGTGAATTATCTCAATAGCAGTGTGAAATAACATAATTGAGCAACTGAAAGGGAGTGCCCAATATTACGACATCATCCATCACCCGCGGCAT<br>TTCCATTTTATTTTGCGAGCGAGCGCACACTTGTGAATTATCTCAATAGCAGTGTGAAATAACATAATTGAGCAACTGAAAGGGAGTGCCCAATATTACGACATCATCCATCACCCGC	-81.5	47	70	17	3730807	100
sxy	(K-12)<br>EC042_1045	TCAACCCTATTTTCGGAAAGAGCCTCGCAAATTTTGTCGTTGGGTGACGGGAAAACATAAATTAATCTTGCCCCTTAAGAATAAGTTGCCTATTTTCGTAGTTAACGGATCCGTTAATGTGAAT<br>TCAACCCTATTTTCGGAAAGAGCCTCGCAAATTTTGTCGTTGGTGACGGGAAAACATAAATTAATCTTGCCCCTTAAGTATAAGTTGCCTATTTTCGTAGTTAACGGATCCGTTAATG	-82.5<br>**(-81.5)**	47<br>**(46)**	70<br>**(69)**	17	1021083<br>**(-1)**	98.4
chbB	(K-12)<br>EC042_1903	TATTTGCCCGAAATGTGAAGAGGGTCATAACCACAGGTCAAGGAGAAACAATTTATAAGGTCAAAGAAATACTATTGCTCAGGTCTATACCGTATACTCCTTTCAGCCACAAAAAAAGTCATGTT<br>TATTTGCCCGAAATGTGAAGAGGGTCATAACCGCAGGTCAAGGAGAGACAATTTATAAGGTCAAAGAAATACTATTGCTCAGGTCTATACCGTATACTCCTTTCAGCCACAAAAAAAG	-83.5	49	72	17	1821726	98.4
cspD	(K-12)<br>EC042_0972	TTCCTGTCAATAGCGTTAACTGCTTCAAATTTTTGATTCATACCTGCCCCTTGTCCCCTGTCAAATGCTTGACGGCTCGCCCTAATTCTCTAAATTGTATTTCTAGAGTTGGCGAGGTTTTGAAC<br>TTCCTGTCAATAGCGTTAACTGCTTCAAATTTTTGATTCATACCTGCCCCCTGGCCCCTGTCAAATGCTTGACGGCTCGCCCTAATTCTCTAAATTGTATTTCTAGAGTTGGCGAGGT	-83.5	48	70	16	922676	98.4
hupBp3	(K-12)<br>EC042_0478	GTGACTGCAAAATAGTGACCTCGCGCAAAATGCACTAATAAAAACAGGGCTGGCAGGCTAATTCGGGCTTGCCAGCCTTTTTTTGTCTCGCTAAGTTAGATGGCGGATCGGGCTTGCCCTTATTA<br>GTGACTGCAAAATAGTGACCTCGCGCAAAATGCACTAATAAAAACAGGGCTGGCAGGCTAATTCGGGCTTGCCAGCCTTTTTTTGTCTCGCTAAGTTAGATGGCGGATCGGGCTTGCC	-83.5	48	71	17	461332	100
sdhC	(K-12)<br>EC042_0739	TAAATGTTGTTATCGTGACCTGGATCACTGTTCAGGATAAAACCCGACAAACTATATGTAGGTTAATTGTAATGATTTTGTGAACAGCCTATACTGCCGCCAGGTCTCCGGAACACCCTGCAATC<br>TAAATGTTGTTATCGTGACCTGGATCACTGTTCAGGATAAAACCCGACAAACTATATGTAGGTTAATTGTAATGATTTTGTGAACGGCCTATACTGCCGCCAGGTCTCCGGAACACCC	-83.5	46	71	19	754958	99.2
spf	(K-12)	AGCTGTCACGTTTTGTGATGGCTATTAGAAATTCCTATGCAACAACTGAAAAAAAATTACAAAAGTGCTTTCTGAACTGAACAAAAAAGAGTAAAGTTAGTCGCGTAGGGTACAGAGGTAAGATG	-84.5	50	72	16	4049899	
serC	(K-12)<br>EC042_0997	AGAGATTCTTTTGTGTGATGCAAGCCACATTTTTGCCCTCAACGGTTTACTCATTGCGATGTGTGTCACTGAATGATAAAACCGATAGCCACAGGAATAATGTATTACCTGTGGTCGCAATCGATTG<br>AGAGATTCTTTTGTGTGATGCAAGCCACATTTTTGCCCTCAACGGTTTTACTCATTGCGATGTGTGTCACTGAATGATAAAACCGATAGCCACAGGAATAATGTATTACCTGTGGTCG	-85.5<br>**(-86.5)**	52<br>**(53)**	74<br>**(75)**	16	957595<br>**(+1)**	99.2
dsdX	(K-12)	TTCACCTTCATTTTTAGATGTAAATCACTCCATTGATGCAATTTACCTCATGTGAAAGGCAAATTTTATCGTTTGTCAGCCTGCGTTGTTTTTTTGTCCAATATCATCAGGTTAATCACAGGGGAAGGT	-87.5	52	75	17	2477821	
casA	(K-12)	GTATGAATATTTTATGTAATAAAATTCATGGTAATTATTATAACTAAAAGTTTCTTTAATAATAAAACGAATAACTTGCAGATTTGAAATGCATGCATTATTGTCTTTAAACAATTCAACACATCTTAATA	-89.5	55	78	17	2884319	
cstAp1	(K-12)<br>EC042_0636	ACTCGGTTAACGGAGTGATCGAGTTAACATTGTTAAGTTAAATATTGGTTTCAACTCCGATTTACATGGTTGCTGTGTTGTTAAATTGTACAAAGATGTTATAGAAACAAAATGTAACATCTCTATGGACA<br>ACTCGGTTAACGGAGTGATCGAGTTAACATTGTTAAGTTAAATATTGGTTTCAACTCCGATTTACATGGTTGCTGTGTTGTTAAATTGCGCAAAGATGTTATAGAAACAAAATGTAAC	-89.5	53	79	20	629855	98.5
nupC	(K-12)	AACATAGCAGAAATGTATGACAGATCACTATTTTTGAAGCCTGTCACAGGACGTCATTATAGTGTGTGTCAGATCTCGTTTTCCTTAACCATGTTACATAGAATGTGCACGGAAATTTAACCTGCCTCATA	-89.5	52	78	20	2513009	
pck	(K-12)<br>EC042_3664	CATTCAGGAATGCGATTCCACTCACAATATTCCCGCCATATAACCAAGATTTAACCTTTTGAGAACATTTTCCACACCTAAAATGCTATTTCTGCGATAATAGCAACCGTTTCGTGACAGGAATCACG<br>CATTCAGGAATGCGATTCCACTCACAATATTCCCGCCACATAAACCAAGATTTAACCTTTTGAGAACATTTTCCACACCTAAAATGCTATTTCTGCGATAATAGCAACCGTTTCG	-89.5<br>**(-90.5)**	52<br>**(53)**	78<br>**(79)**	20	3532679<br>**(+1)**	98.4
uidA	(K-12)<br>EC042_1785	TTTTAAAGATTAATGCGATCTATATCACGCTGTGGGTATTGCAGTTTTTGGTTTTTTGATCGCGGTGTCAGTTCTTTTTATTTCCATTTCTCTTCCATGGGTTTCTCACAGATAACTG<br>TTTTAAAGATTAATGCGATCTATATCACGCTGTGGGTATTGCAGTTTTTGGTTTTTTGATCGCGGTGTCAGTTCTTTTTATTTCCATTTCTCTTCCATGGGTTTCTCACAGATAACTG	-89.5	51	75	18	1696152	100
araJ	(K-12)<br>EC042_0428	CAGCAAACTGGAAAGTACGTTTGCAGTGAAATAACTATTCAGCAGGATAATGAATACAGAGGGGCGAATTATCTCTTGGCCTTGCTGGTCGTTATCCTGCAAGCTATCACTTTATTGGCTACGGTGATTGGT<br>CAGCAAACTGGAAAGTGTGTTTGCAGTGAAATAACTATTCAGCAGGATAATGAATACAGAGGGGCGAATTATCTCTTGGCCTCGCTGGTCGTTATCCTGCAAGCTATCACTTTATTGG	-90.5	55	79	18	412529	97
aspA	(K-12)<br>EC042_4615	GTAATCCCAAAGCGGTGATCTATTTCACAAATTAATAATTAAGGGGTAAAAACCGACACTTAAAGTGATCCAGATTACGGTAGAAATCCTCAAGCAGCATATGATCTCGGGTATTCGGTCGATGCAGGGGAT<br>GTAATCCCAAAGCGGTGATCTATTTCACAAATTAATAATTAAGGGGTAAAAACCGACACTTAAAGTGATCCAGATTACGGTAGAAATCCTCAAGCAGCATATGATCTCGGGTATTCGG	-90.5	54	79	19	4368432	100
glpA	(K-12)	TTCATAAATTAAATGTGAATTGCCGCACACATTATTAAATAAGATTTACAAAATGTTCAAAATGACGCATGAAATCACGTTTCACTTTCGAATTATGAGCGAATATGCGCGAAATCAAACAATTCATGTTTT	-90.5	55	80	19	2352583	
ansBp2	(K-12)<br>EC042_3164	TAATTCTTCGTTTTGTTACCTGCCTCTAACTTTGTAGATCTCCAAAATATATTCACGTTGTAAATTGTTTAACGTCAAATTTCCCATACAGAGCTAAGGGATAATGCGTAGCGTTCACGTAACTGGAGGAATG<br>TAATTCTTCGTTTTGTTACCTGCCTCTAACTTTGTAGATCTCCAAAATATATTCACGTTGTAAATTGTTTAACGTCAAATTTCCCATACAGAGCTAAGGGATAATGCGTAGCGTTCAC	-91.5	53	79	20	3100751	100
glpT	(K-12)	TTCGAAAGTGAAACGTGATTTCATGCGTCATTTTGAACATTTTGTAAATCTTATTTAATAATGTGTGCGGCAATTCACATTTAATTTATGAATGTTTTCTTAACATCGCGGCAACTCAAGAAACGGCAGGTTC	-91.5	53	80	21	2352451	
cdd	(K-12)	ATGGGCTAAAATTTGCGATGCGTCGCGCATTTTTGATGTATGTTTCACGCGTTGCATAATTAATGAGATTCAGATCACATATAAAGCCACAACGGGTTCGTAAACTGTTATCCCATTACATGATTATGAGGCAA	-92.5	53	80	21	2231819	
manX	(K-12)	CTTTGCAAACGAATGTGACAAGGATATTTTACCTTTCGAAATTTCTGCTAATCGAAAGTTAAATTACGGATCTTCATCACATAAAATAATTTTTTCGATATCTAAAATAAATCGCGAAACGCAGGGGTTTTTGG	-92.5	55	82	21	1901933	
nupG	(K-12)	TCAGGGGCAAAAATGTTATCCACATCACAATTTCGTTTTGCAAATTGGGAATCTTTGCAATTATTTGCCACAGGTAACAAAAAACCAGTCCGCGAAGTTGATAGAATCCCATCATCTCGCACGGTCAAATGTGC	-92.5	54	81	21	3105651	
ompF	(K-12)<br>EC042_1020	AAGTTCCTTAAATTTTACTTTTGGTTACATATTTTTTCTTTTTGAAACCAAATCTTTATCTTTGTAGCACTTTCACGGTAGCGAAACGTTAGTTTGAATGGAAAGATGCCTGCAGACACATAAAGACACCAAAC<br>AAGTTCCTTAAATTTTACTTTTGGTTACATATTTTTTCTTTTTGAAACCAAATCTTTATCTTTGTAGCACTTTCACGGTAGCGAAACGTTAGTTTGAATGGAAAGATGCCTGCAGACA	-92.5	57	81	18	987092	100
rhaB	(K-12)<br>EC042_4277	CAATTCAGCAAATTGTGAACATCATCACGTTCATCTTTCCCTGGTTGCCAATGGCCCATTTTCCTGTCAGTAACGAGAAGGTCGCGAATTCAGGCGCTTTTTAGACTGGTCGTAATGAAATTCAGCAGGATCAC<br>CAATTCAGCAAATTGTGAACATCATCACGTTCATCTTTCCCTGGTTGCCAATGGCCCATTTTCCTGTCAGTAACGAGAAGGTCGCGAAGTCAGGCGCTTTTTAGACTGGTCGTAATGA	-92.5	60	81	15	4097472	99.3
rhaS	(K-12)<br>EC042_4278	GCTCACCGCATTTCCTGAAAATTCACGCTGTATCTTGAAAAATCGACGTTTTTACGTGGTTTTCCGTCGAAAATTTAAGGTAAGAACCTGACCTCGTGATTACTATTTCGCCGTGTTGACGACATCAGGAGGC<br>GCTTACCGCATTTCCTGAAAATTCACGCTGTATCTTGAAAAATCGACGTTTTTACGTGGTTTTCCGTCGAAAATTTAAGGTAAGAACCTGACCTCGTGATTACTATTTCGCCGTGTT	-92.5	60	81	15	4097711	99.2

[a]Promoter sequences are derived from RegulonDB(11) and EcoCyc(13). The annotated CRP site is highlighted yellow, the -35 element is highlighted grey, the -10 element is highlighted cyan, and the transcription start site is highlighted green.

[b]Differences in the promoter sequence found in the 042 genome are in given in parenthesis and are in bold.

[c]The percentage of nucleotide identity across the given sequence.

Supplementary material references

1. Nataro JP, Deng Y, Cookson S, Cravioto A, Savarino SJ, Guers LD, et al. Heterogeneity of enteroaggregative Escherichia coli virulence demonstrated in volunteers. J Infect Dis. 1995 Feb;171(2):465–8.

2. Casadaban MJ, Cohen SN. Analysis of gene control signals by DNA fusion and cloning in Escherichia coli. J Mol Biol. 1980 Apr;138(2):179–207.

3. Busby S, Kotlarz D, Buc H. Deletion mutagenesis of the Escherichia coli galactose operon promoter region. J Mol Biol. 1983 Jun 25;167(2):259–74.

4. Lodge J, Fear J, Busby S, Gunasekaran P, Kamini NR. Broad host range plasmids carrying the Escherichia coli lactose and galactose operons. FEMS Microbiol Lett. 1992 Aug 15;74(2–3):271–6.

5. Islam MS, Pallen MJ, Busby SJW. A cryptic promoter in the LEE1 regulatory region of enterohaemorrhagic Escherichia coli: promoter specificity in AT-rich gene regulatory regions. Biochem J. 2011 Jun 15;436(3):681–6.

6. Kolb A, Kotlarz D, Kusano S, Ishihama A. Selectivity of the Escherichia coli RNA polymerase E sigma 38 for overlapping promoters and ability to support CRP activation. Nucleic Acids Res. 1995 Mar 11;23(5):819–26.

7. West D, Williams R, Rhodius V, Bell A, Sharma N, Zou C, et al. Interactions between the Escherichia coli cyclic AMP receptor protein and RNA polymerase at class II promoters. Mol Microbiol. 1993 Nov;10(4):789–97.

8. Zhang Y, Liu T, Meyer CA, Eeckhoute J, Johnson DS, Bernstein BE, et al. Model-based Analysis of ChIP-Seq (MACS). Genome Biology. 2008 Sep 17;9(9):R137.

9. Grainger DC, Hurd D, Harrison M, Holdstock J, Busby SJW. Studies of the distribution of Escherichia coli cAMP-receptor protein and RNA polymerase along the E. coli chromosome. Proc Natl Acad Sci U S A. 2005 Dec 6;102(49):17693–8.

10. Bailey TL, Johnson J, Grant CE, Noble WS. The MEME Suite. Nucleic Acids Res. 2015 Jul 1;43(W1):W39-49.

11. Salgado H, Gama-Castro S, Lara P, Mejia-Almonte C, Alarcón-Carranza G, López-Almazo AG, et al. RegulonDB v12.0: a comprehensive resource of transcriptional regulation in E. coli K-12. Nucleic Acids Res. 2024 Jan 5;52(D1):D255–64.

12. Chaudhuri RR, Sebaihia M, Hobman JL, Webber MA, Leyton DL, Goldberg MD, et al. Complete Genome Sequence and Comparative Metabolic Profiling of the Prototypical Enteroaggregative Escherichia coli Strain 042. PLoS One. 2010 Jan 20;5(1):e8801.

13. Keseler IM, Bonavides-Martínez C, Collado-Vides J, Gama-Castro S, Gunsalus RP, Johnson DA, et al. EcoCyc: a comprehensive view of Escherichia coli biology. Nucleic Acids Res. 2009 Jan;37(Database issue):D464-470.
